# Spatiotemporal analysis of substance use disorder mortality in the United States: an observational study of emerging hotspots and vulnerable populations (2005–2020)

**DOI:** 10.1016/j.lana.2025.101075

**Published:** 2025-04-04

**Authors:** Santiago Escobar, Neil J. MacKinnon, Preshit Ambade, Zach Hoffman, Diego F. Cuadros

**Affiliations:** a Digital Epidemiology Laboratory, Digital Futures, University of Cincinnati, Cincinnati, OH, USA; b College of Medicine, Central Michigan University, Mount Pleasant, MI, USA

**Keywords:** Spatiotemporal clustering, Substance use disorder mortality, Overdose, Addiction, Scan statistics, Spatial epidemiology, Longitudinal studies

## Abstract

**Background:**

The escalating substance use disorder (SUD) crisis in the contiguous United States (US), with rising mortality since 1999, necessitates a spatiotemporal analysis to identify high-risk areas and vulnerable populations. This study examines the geospatial distribution and clustering patterns of SUD mortality, assessing disparities by race and urban-rural status.

**Methods:**

We analyzed county-level ecological data on SUD-related deaths from the Centers for Disease Control and Prevention (CDC) from 2005 to 2020. Using spatial scan statistics, we identified significant clusters of elevated SUD mortality and assessed shifts over time. The analysis stratified results by race (White and Black subpopulations) and urban-rural classification to explore disparities.

**Findings:**

Among 3142 U.S. counties, we identified 27 significant spatiotemporal clusters of elevated SUD mortality, primarily emerging post-2013 and persisting until 2020. The epidemic's epicenter shifted from the western to the eastern U.S. around 2016. Clusters in the White population (n = 26) had an estimated mortality rate of 28.42 per 100,000 person-years (95% confidence interval [CI]: 28.30–28.54), compared to 14.83 per 100,000 person-years (95% CI: 14.74–14.92) outside clusters. For the Black population (n = 17), the mortality rate was 33.16 per 100,000 person-years (95% CI: 32.80–33.51) within clusters, versus 13.36 per 100,000 person-years (95% CI: 13.14–13.59) outside. Clusters in the Black population emerged later, mostly after 2013, while White clusters followed a pattern similar to the general population. The urban SUD mortality rate was 1.30 per 10,000 per year, while the rural mortality rate was 1.03 per 10,000 per year. Within clusters, urban counties had a mortality rate of 1.61 per 10,000, compared to 0.97 per 10,000 outside. Rural counties had 1.43 per 10,000 in clusters, while non-clustered rural areas had 0.81 per 10,000.

**Interpretation:**

The shifting geographic and racial patterns of SUD mortality underscore the need for targeted, region-specific interventions. The increasing impact on Black populations and urban centers in the East highlights the importance of equitable access to treatment and harm reduction services. Real-time surveillance and tailored urban-rural strategies are essential to mitigate the evolving crisis.

**Funding:**

None.

**Research in context**

**Evidence before this study**

We conducted a comprehensive literature review to assess the current state of research on the spatiotemporal dynamics of substance use disorder (SUD) in the US. Sources included PubMed, Google Scholar, and relevant epidemiological and public health journals. Our search criteria focused on studies published between January 2000 and December 2022, examining the spatial distribution and temporal trends of SUD-related mortality, with particular attention to the impact on different racial and demographic groups. Key references included analyses of opioid overdose trends, racial disparities in SUD outcomes, and urban-rural differences in substance use patterns. We identified significant gaps in understanding the evolving geographical epicenter of the SUD epidemic and the need for fine-scale temporal and spatial analyses to inform public health interventions.

**Added value of this study**

This study advances the existing evidence by providing a detailed spatiotemporal analysis of SUD-related mortality in the US from 2005 to 2020. Applying spatial scan statistics, we identified 27 significant spatiotemporal clusters of elevated mortality rates, highlighting a dynamic shift in the epicenter of the epidemic from the western to the eastern US around 2016. Our findings underscore significant racial disparities, with the black subpopulation emerging as a newly vulnerable group with increasing mortality rates since 2013. Furthermore, we documented distinct urban-rural variations, particularly noting that rural areas in the West are more likely to form clusters compared to their eastern counterparts. These insights offer a granular understanding of the progression of the epidemic and the demographic groups most at risk, informing targeted public health strategies.

**Implications of all the available evidence**

The evolving and highly dynamic nature of the SUD epidemic highlights the need for continuous surveillance and adaptive public health responses. As the epidemic shifts to the eastern U.S. and disproportionately impacts Black populations, interventions must be region-specific and equity-focused. Expanding harm reduction and treatment services in newly affected urban areas is critical, alongside addressing systemic healthcare disparities and improving culturally competent care for Black communities. The distinct urban-rural disparities call for tailored strategies, particularly in rural areas with limited healthcare access and high prescription opioid use. Integrating real-time mortality tracking and advanced surveillance systems will enhance the timely identification of emerging hotspots, allowing public health efforts to remain proactive and responsive to the shifting epidemic landscape.

## Introduction

Substance use disorder (SUD) is defined as a treatable mental disorder that affects a person's ability to control their use of substances, understood as compounds that can potentially be abused for recreational purposes due to their psychoactive nature.^1^ This condition has been rising for over three decades, claiming over 900,000 lives in the United States (US) since 1999 and constituting a national epidemic.^2^ Deaths from SUD have increased more than any other cause of death in this period, with SUD-related deaths surpassing motor vehicle traffic as the most frequent unintentional injury-related death cause in 2012 and staying on top ever since.^3^ The spatial distribution of the SUD epidemic has shown a heterogeneous pattern, with some areas exhibiting increased risk. The West and Midwest regions of the US have shown clusters of SUD mortality,^4^ with temporal and social dynamics showing regional differences accounting for the local variation of the national epidemic.

Temporarily, there are four distinct but overlapping waves of the epidemic.^5^ The first wave, characterized by prescription opioids, occurred between 2000 and 2016. A rise in heroin-related deaths marked the second wave, beginning in 2007 and surpassing prescription-related deaths in 2015. The third wave is linked to synthetic opioids, which exhibited a steady increase from 2013 to 2018. The fourth wave is associated with a rise in polydrug use following the COVID-19 pandemic and could be considered to still be ongoing.

The racial differences are prominent in SUDs, with higher overall mortality among whites but with a sharp rise among black males post-2014.^6^ In addition, the urban-rural divide has also created geographical differences: rural areas present a higher percentage of the white population, higher unemployment rates, and a greater percentage of specialized opioid prescribers, such as surgeons and oncologists,^7^ while lacking resources to treat SUD.^8^ These differences observed in the SUD epidemic can be attributed to supply and demand: the availability of certain substances over limited geographical areas may have caused variations in the observed mortality trends over time.^9^

Previous studies,^4^ conducted a spatial analysis of the data up to 2017, highlighting geospatial hotspots emphasizing vulnerable populations. Extending this analysis further, this research seeks to explore the temporal dynamics of the spatial structure of the SUD crisis by examining the spatiotemporal dynamics of SUD mortality rates, including data until 2020.

The aim of this study is to analyze the spatiotemporal dynamics of SUD mortality in the U.S. from 2005 to 2020, identifying evolving geographic patterns and racial disparities. Using spatiotemporal clustering analysis, we seek to detect high-risk areas, characterize emerging local micro-epidemics, and assess the shifting epicenter of the crisis over time. This study provides insights into how SUD mortality has evolved across racial groups and geographic landscapes, informing targeted public health interventions.

## Methods

### Study design

Institutional review board approval and informed consent were not necessary for this cross-sectional study because all data were deidentified and publicly available (Common Rule 45 CFR §46). This study follows the Strengthening the Reporting of Observational Studies in Epidemiology (STROBE) reporting guideline. A longitudinal ecological study was conducted to identify the spatiotemporal dynamics of SUD mortality across the contiguous U.S from 2005 to 2020. By employing retrospective spatiotemporal scan statistics, this study sought to identify the spatial and temporal structure followed by the SUD epidemic, analyzing the behavior of SUD—related deaths across the general population, as well as across the White, Black, Rural, and Urban subpopulations.

### Data sources

SUD mortality data was collected from the US Vital Statistics System restricted-use micro-data mortality files for the period of January 2005 to December 2020,^10^ the filtered data included date and county of deaths coded as unintentional acute poisoning events, decedent's demographic characteristics (sex and race) and the International Classification of Diseases, 10th Revision (ICD-10) code for the cause of death.^11^ Individuals aged 5–84 years were included, and drug overdoses were identified as those with ICD-10 codes indicating unintentional substance poisoning (cause of death codes: X40, X41, X42, X43, X44) to estimate SUD mortality rates. These ICD-10 codes included deaths caused by the following substances: heroin, methadone, cocaine, other opioids, synthetic narcotics, and unspecified narcotics. Chronic deaths related to the long-term effects of substance use (e.g., liver disease or cardiovascular complications) were not included in the scope of this analysis. This approach ensures that the study focuses specifically on the acute mortality burden directly attributable to SUD. Using this definition, the sample size for this study included all SUD mortality cases reported in the contiguous United States between 2005 and 2020, totaling 665,341 deaths. This comprehensive approach ensures that the analysis captures the full extent of the epidemic's dynamics, maximizing the statistical power necessary to detect significant spatiotemporal clusters.

Population at risk was estimated by using the latest county-level population estimates from the U.S Census Population and Housing Unit Estimates Tables for each of the years of our study period.^12^ Data was filtered by subpopulation, obtaining yearly population estimates by demographic characteristics (sex and race). Further, counties were classified as urban or rural following the National Center for Health Statistics (NCHS) 2013 Urban-Rural classification schemes^13^: Large central metro, Large fringe metro, Medium metro, and Small metro counties were considered urban, while Micropolitan and Noncore counties were considered rural.

To delineate the heterogeneity in the estimates, county-level mortality rates are reported for the general population, encompassing the three racial categories reported in the mortality files of White, Black, and Others. Further, separate estimates for White, Black, Urban, and Rural subpopulations are also reported.

### Spatiotemporal clustering analysis

A county-level spatiotemporal clustering analysis was conducted to identify geographical clusters of high numbers of SUD-related deaths that persist for at least two or more years using Kulldorff's spatial scan statistics^14^ implemented in the SaTScan software. The unit of analysis in this study is the county, as mortality data were aggregated at the county level. Clusters represent groups of neighboring counties with significantly elevated SUD mortality during specific time periods.

The analysis was performed for the general population, as well as for the White and Black subpopulations. Scan statistics are widely used for cluster detection in epidemiology,^15^, ^16^, ^17^, ^18^ social sciences,^19^ crime mapping,^20^ and, very recently, in mental health,^21^ among other applications. We applied a Poisson likelihood function, which is suitable for count data, to model the occurrence of SUD mortality events in relation to the population at risk within each county. To estimate statistical significance of the clusters detected, SaTScan estimates p-values using a Monte Carlo hypothesis testing approach within the framework of the spatial scan statistic. Specifically, for the discrete Poisson model, SaTScan first computes the likelihood ratio test (LRT) statistic for each scanning window, comparing the observed number of cases within the window to the expected number under the null hypothesis of spatial randomness. The window with the maximum likelihood ratio is identified as the most likely cluster. To assess statistical significance, SaTScan generates multiple random datasets under the null hypothesis by distributing cases according to a Poisson process that maintains the overall distribution of the population at risk. For each simulated dataset, the likelihood ratio is recalculated, forming a reference distribution of test statistics under the null hypothesis. The p-value for an observed cluster is then estimated as the proportion of Monte Carlo replicates that yield a likelihood ratio at least as large as that of the observed cluster. This approach accounts for multiple testing inherent in scanning multiple potential cluster locations and sizes. Clusters with p-values below α = 0.05, are considered statistically significant. A detailed description of the spatial scan statistics is provided elsewhere.^14^^,^^17^ Likewise, a detailed description of the Satscan methodology implemented and the assumptions for the Poisson model, confidence interval estimations, and sensitivity analysis of model assumptions can be found in Supplementary Methods and Supplementary Figure S1 in Supplementary Materials.

After a cluster was identified, the strength of the clustering was estimated using the relative risk (RR) of cases within the cluster versus outside the cluster. The number of SUD-related deaths from the complete dataset (2005–2020) was analyzed at the county level using Kulldorff's spatial scan statistics with a Poisson model with the overall population size by county included as an offset. SUD mortality rates are reported as the number of deaths per 10,000 people. Sensitivity analyses were performed to assess the robustness of the findings, including variations in Monte Carlo replications and outlier exclusions, and are detailed in Supplementary Methods in Supplementary Materials.

### Analyzing the urban and rural divide in the epidemic

To assess the distribution of the epidemic across urban and rural areas, bivariate choropleth maps were produced for the urban/rural status of a county and if it was classified as a cluster for the general population at some point in the study period. To illustrate the statistical relationship established between these variables and their differences in the East and West of the country, proportionate bar graphs were produced using the R package ggplot2.^22^ Lastly, contingency tables were created for the urban/rural status of a county and whether it was classified as a cluster for the general population at some point in the study period, these tables were used to estimate the RR for a rural county being classified as a cluster compared to an urban county. RRs for rural counties compared to their urban counterparts were estimated for the whole country, as well as counties to the East and to the West of the country.

### Temporal dynamics across subpopulations

Mean mortality values were estimated across the country by year for the general population, as well as for the White, Black, Urban, and Rural subpopulations. The mean mortality estimates were used to produce temporal line graphs representing the variability in mortality trends across time by subpopulation.

### Role of the funding source

This research received no specific grant from any funding agency in the public, commercial, or not-for-profit sectors. The authors had full access to all data in the study and had final responsibility for the decision to submit for publication.

## Results

### General results

Deaths by unintentional drug overdose in the contiguous U.S. from 2005 to 2020 resulted in 665,341 cases. The estimated average SUD mortality rate during this period in the total population was 13.36 per 100,000 person-years (95% CI −13.32 to 13.40). The state with the highest mortality rate during this period was West Virginia, with a mortality rate of 28.5 per 100,000 person-years (95% CI 27.64–28.88), followed by Kentucky (22.26 per 100,000 person-years; 95% CI 21.91–22.61), New Mexico (20.69 per 100,000 person-years; 95% CI 20.21–21.18), District of Columbia (20.64 per 100,000 person-years; 95% CI 19.77–21.55), and Delaware (19.53 per 100,000 person-years; 95% CI 18.83–20.26). The estimated SUD mortality rate in the White subpopulation was 14.11 per 100,000 person-years (95% CI 14.06–14.16), whereas the mortality rate in the Black subpopulation was 12.54 per 100,000 person-years (95% CI 12.46–12.62). Supplementary Table S1 in Supplementary Materials summarizes General estimations for the total population and the Black and White populations, and Supplementary Figure S2 illustrates the temporal trend of the mortality rate for the total, Black and White populations from 2005 to 2020.

### Spatiotemporal clustering analysis for the general population

**Fig. 1:**
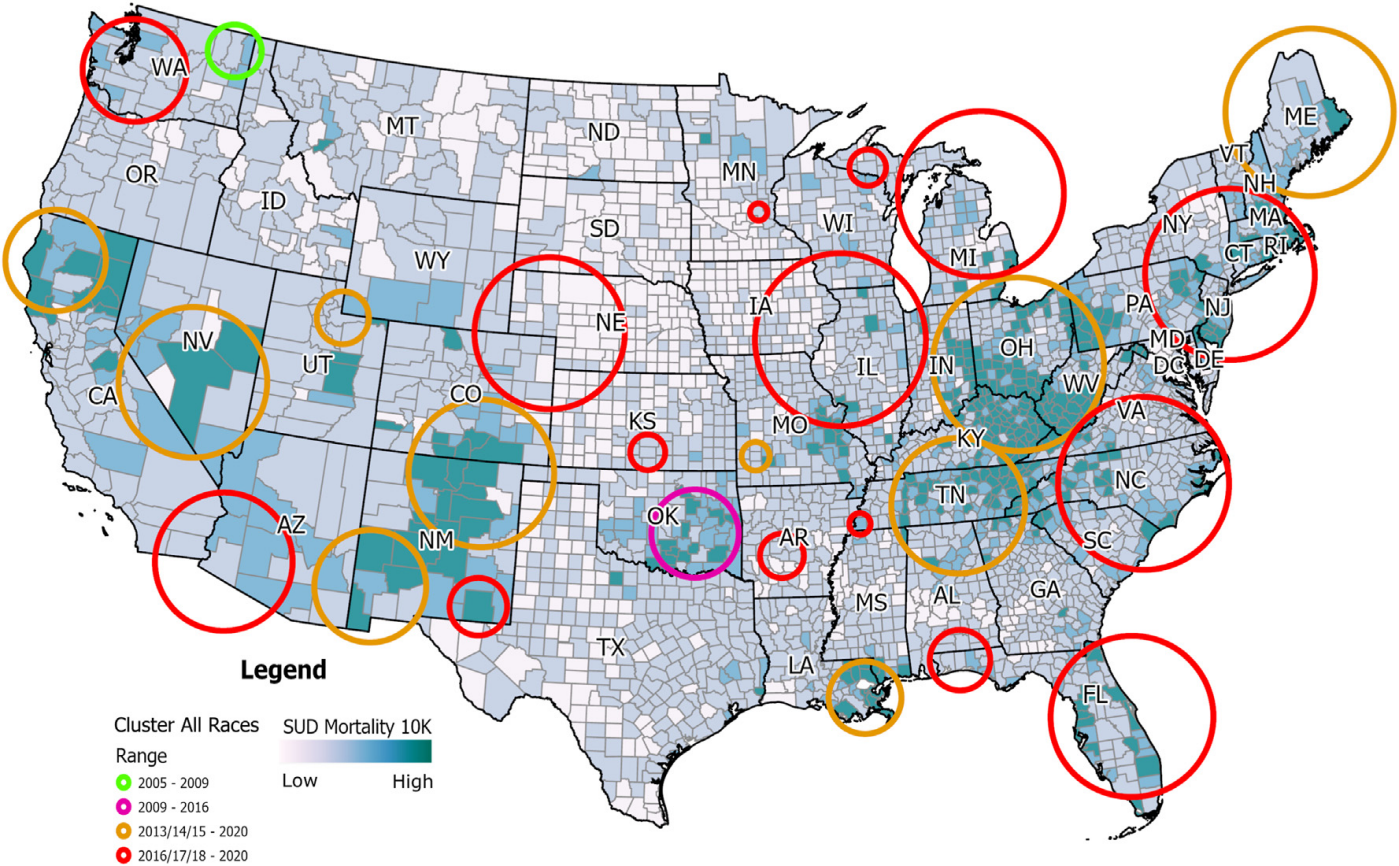
**Spatiotemporal clustering of SUD-related deaths in the contiguous United States (2005–2020)***.* This map shows significant spatiotemporal clusters of SUD-related deaths in the contiguous United States from 2005 to 2020, identified using Kulldorff's spatial scan statistics. Early clusters appeared in Washington (2005–2009) and Oklahoma (2009–2016). From 2013 onward, clusters emerged in the western US, shifting to the eastern US post-2016. The estimated mortality rate within the clusters was 25.94 per 100,000 person-years (95% CI 25.83–26.04) compared with a mortality rate of 13.61 per 10,000 person-years (95% CI 13.53–13.69) estimated in the areas outside of the identified clusters.

Spatiotemporal SaTScan analysis identified 27 statistically significant spatiotemporal clusters that started emerging in 2005 (Fig. 1). Summary of the description of the clusters identified, including p values, are reported in Supplementary Table S2 in Supplementary Materials. The estimated mortality rate within the clusters was 25.94 per 100,000 person-years (95% CI 25.83–26.04) compared with a mortality rate of 13.61 per 10,000 person-years (95% CI 13.53–13.69) estimated in the areas outside of the identified clusters. One cluster emerged in the state of Washington in 2005, lasting until 2008 (average mortality rate 18.25 per 100,000 person-years; 95% CI 14.67–24.83), a second early cluster emerged in the state of Oklahoma in 2009, lasting until 2016 (average mortality rate 18.22 per 100,000 person-years; 95% CI 16.62–19.81). The 25 remaining clusters are divided into two main temporal groups: ten clusters starting from 2013 to 2015 and lasting until the end of the study period, and 15 clusters starting from 2016 to 2018 and lasting until the end of the study period. Broadly, most of the clusters emerging before 2016 were in the western part of the country, whereas most of the clusters emerging after 2016 were in the eastern part of the country. Supplementary Table S2 in Supplementary Materials summarizes the main analysis outcomes from SaTScan for the general population.

### Spatiotemporal clustering analysis for the subpopulations

**Fig. 2:**
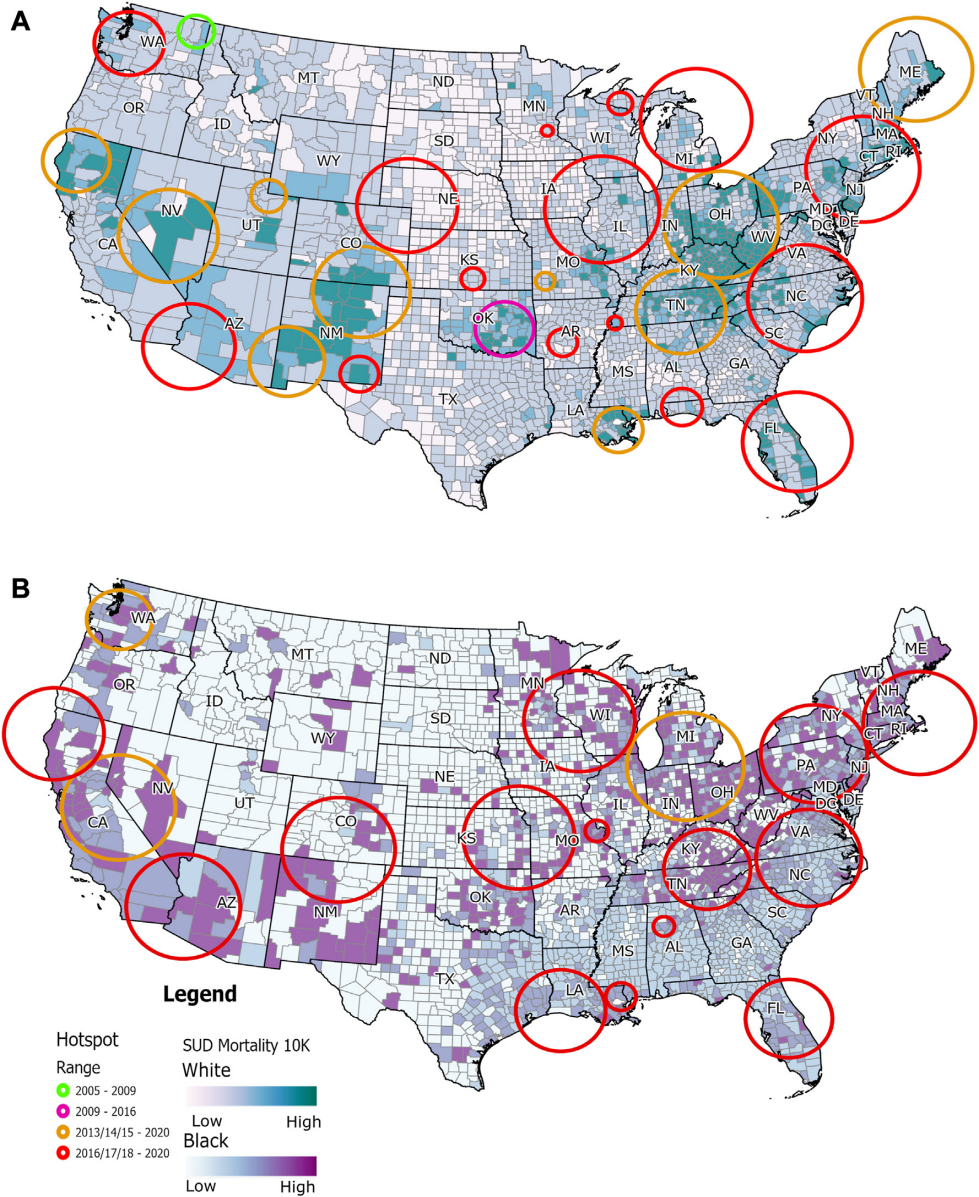
**Spatiotemporal clusters of SUD mortality by race in the US (2005–2020)***.* This figure displays spatiotemporal clusters of SUD-related deaths by race in the contiguous United States from 2005 to 2020. A) Clusters for the White subpopulation. A total of 26 significant clusters were identified. Early clusters appeared in Washington (2005–2009) and Oklahoma (2009–2016), with additional clusters emerging in California. Post-2013, clusters proliferated in the western US, with a shift to the eastern US post-2016. The mortality rate within clusters for the White population 28.42 per 100,000 person-years (95% CI 28.30–28.54) compared with a mortality rate of 14.83 per 100,000 person-years (95% CI 14.74–14.92) estimated in the areas outside of the identified clusters. B) Clusters for the Black subpopulation. Seventeen significant clusters were identified, all emerging between 2013 and 2018 and continuing until 2020. These clusters were predominantly located in the eastern US. The mortality rate estimated inside of the counties identified as clusters was 33.16 per 100,000 person-years (95% CI 32.80–33.51), while in the counties outside of clusters the estimated mortality rate was 13.36 per 100,000 person-years (95% CI 13.14–13.59). This reflects a later but rapid emergence of clusters in the Black subpopulation compared to the White subpopulation.

Spatiotemporal clusters of high SUD mortality were also detected for the White and Black subpopulations, using the same analysis and parameters. For the White subpopulation, 26 significant clusters were reported. Summary of the description of the clusters identified, including p values, are reported in Supplementary Table S3 in Supplementary Materials. The spatial distribution of these clusters exhibits similar structure as observed for the general population (Fig. 2A). The estimated mortality rate within the clusters was 28.42 per 100,000 person-years (95% CI 28.30–28.54) compared with a mortality rate of 14.83 per 100,000 person-years (95% CI 14.74–14.92) estimated in the areas outside of the identified clusters. One cluster emerged in the state of Washington between the years 2005 and 2008 (average mortality rate of 19.43 per 100,000 person-years; 95% CI 15.53–23.32), two more early clusters showed up in the states of California and Oklahoma from 2009 to 2016 (average mortality rate of 25.83 and 20.37 per 100,000 person-year respectively). Eleven clusters starting from 2013 to 2015 and lasting until the end of the study period were reported, and 12 clusters starting from 2016 to 2018 and lasting until the end of the study period. Similar to the results reported for the general population, most of the clusters pre-2016 emerged in the western part of the country, whereas the clusters post-2016 emerged in the eastern part of the country. Supplementary Table S3 in Supplementary Materials summarizes the main analysis outcomes from SaTScan for the White population.

For the Black subpopulation, 17 significant clusters were identified, all of which started between 2013 and 2018 and continued to 2020 (Fig. 2B). Summary of the description of the clusters identified, including p values, are reported in Supplementary Table S4 in Supplementary Materials. The mortality rate estimated inside of the counties identified as clusters was 33.16 per 100,000 person-years (95% CI 32.80–33.51), while in the counties outside of clusters the estimated mortality rate was 13.36 per 100,000 person-years (95% CI 13.14–13.59). Only three clusters were reported between 2013 and 2015, while the remaining 14 clusters were reported as emerging after 2016. These SUD clusters for Black subpopulation were predominantly located in the eastern part of the country. Supplementary Table S4 in Supplementary Materials summarizes the main analysis outcomes from SaTScan for the Black population.

Supplementary Figure S3 in Supplementary Materials summarizes the notable temporal dynamics and racial disparities in SUD-related mortality, with high-risk clusters emerging later among the Black population compared to the general and White populations. Starting around 2015, high-risk clusters in Black communities show elevated RR, often exceeding those seen in earlier clusters among other groups. This delayed but intensified emergence suggests a recent and concentrated impact of the SUD epidemic in Black communities, indicating potentially heightened vulnerability or exacerbated risk factors in recent years.

We assessed the stability of cluster detection using different Monte Carlo replications (999, 4999, and 9999). Clusters for the general (0% misclassification) and Black populations were stable across all levels, indicating robust results. The White population showed minor variation, with a southeastern New Mexico cluster appearing only at the standard level (999 replications), resulting in a slightly higher misclassification rate of 0.3%.

An additional outlier exclusion analysis, which removed counties in the 98th percentile of SUD-related deaths, demonstrated consistent cluster patterns across populations, with only minor shifts. Misclassification rates remained low at 15% for the general population and were 28% and 36% for White and Black populations, respectively, indicating expected variability without systematic bias. Sensitivity analyzes conducted are summarized in Supplementary Tables S4–S7 and Supplementary Figures S4–S9 in Supplementary Materials.

### SUD mortality in urban and rural areas

**Fig. 3:**
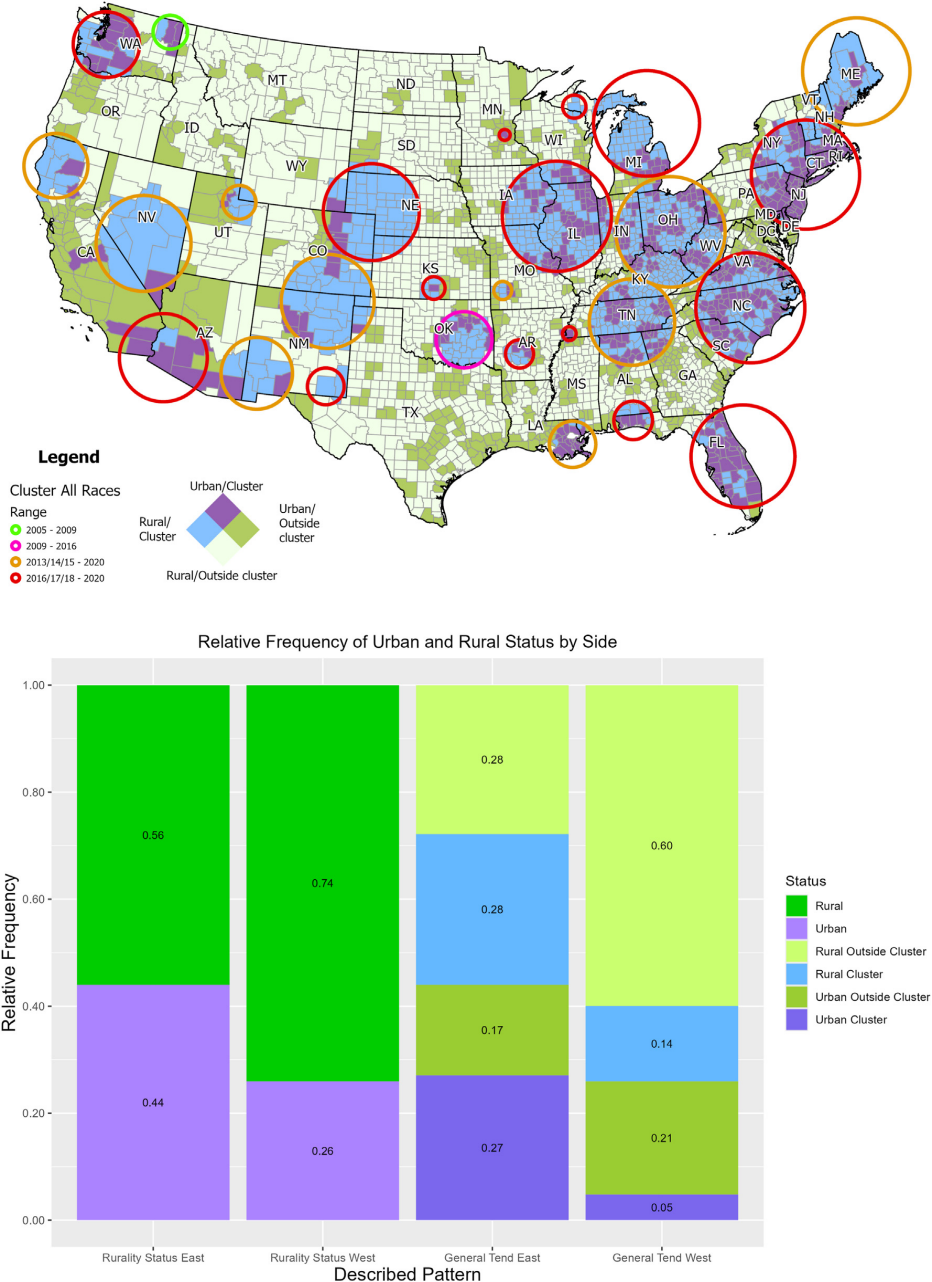
**Urban-rural differences in SUD mortality clusters in the US (2005–2020)***.* This figure illustrates the urban-rural differences in SUD-related mortality clusters in the contiguous United States from 2005 to 2020. A) Bivariate choropleth map showing urban and rural counties classified as clusters. Urban counties are predominantly found in clusters in the eastern US, whereas clusters in the western US are mostly rural. The map highlights the spatial distribution of high-risk areas, with urban areas showing higher cluster prevalence in the East and rural areas in the West. B) Bar chart depicting the proportion of urban and rural counties within identified clusters across the US. The chart demonstrates a higher likelihood of rural counties being classified as clusters in the western US, while urban counties are more prevalent in clusters in the eastern U.S.

The estimated average SUD mortality rate for the study period was 1.30 per 10,000 people per year in the urban areas, compared to 1.03 per 10,000 people per year in the rural areas. The average mortality rate in urban counties classified as clusters was 1.61 per 10,000 people per year, while in urban counties not classified as clusters it was 0.97 per 10,000 people per year. For rural counties classified as clusters the average mortality rate was 1.43 per 10,000 people per year, while rural counties not classified as clusters had an average mortality rate of 0.81 per 10,000 people per year. It is important to note that due to the nature of the analysis, clusters can contain both urban and rural counties, but as it can be observed in Fig. 3, clusters located to the West of the country are predominantly rural, whereas to the East of the country the proportion of urban and rural counties inside of clusters is relatively even.

The analysis of the data for the country in general revealed a RR of 0.73 (95% CI 0.65–0.81) for belonging to a cluster in rural counties compared to urban counties: Rural counties were at decreased risk of being classified as a cluster compared to their urban counterparts, which implies that the presence of urban counties within clusters was greater than that of rural counties. In terms of the urban/rural concentration of counties within a cluster, the East and West regions of the US showed a contrasting picture: Rural counties from the East were less likely to be in a SUD cluster compared to their urban counterparts (RR 0.82 95% CI 0.73–0.92), whereas in the west, the likelihood was higher (RR 1.03 95% CI 0.76–1.40).

### Temporal dynamics by subpopulation

**Fig. 4:**
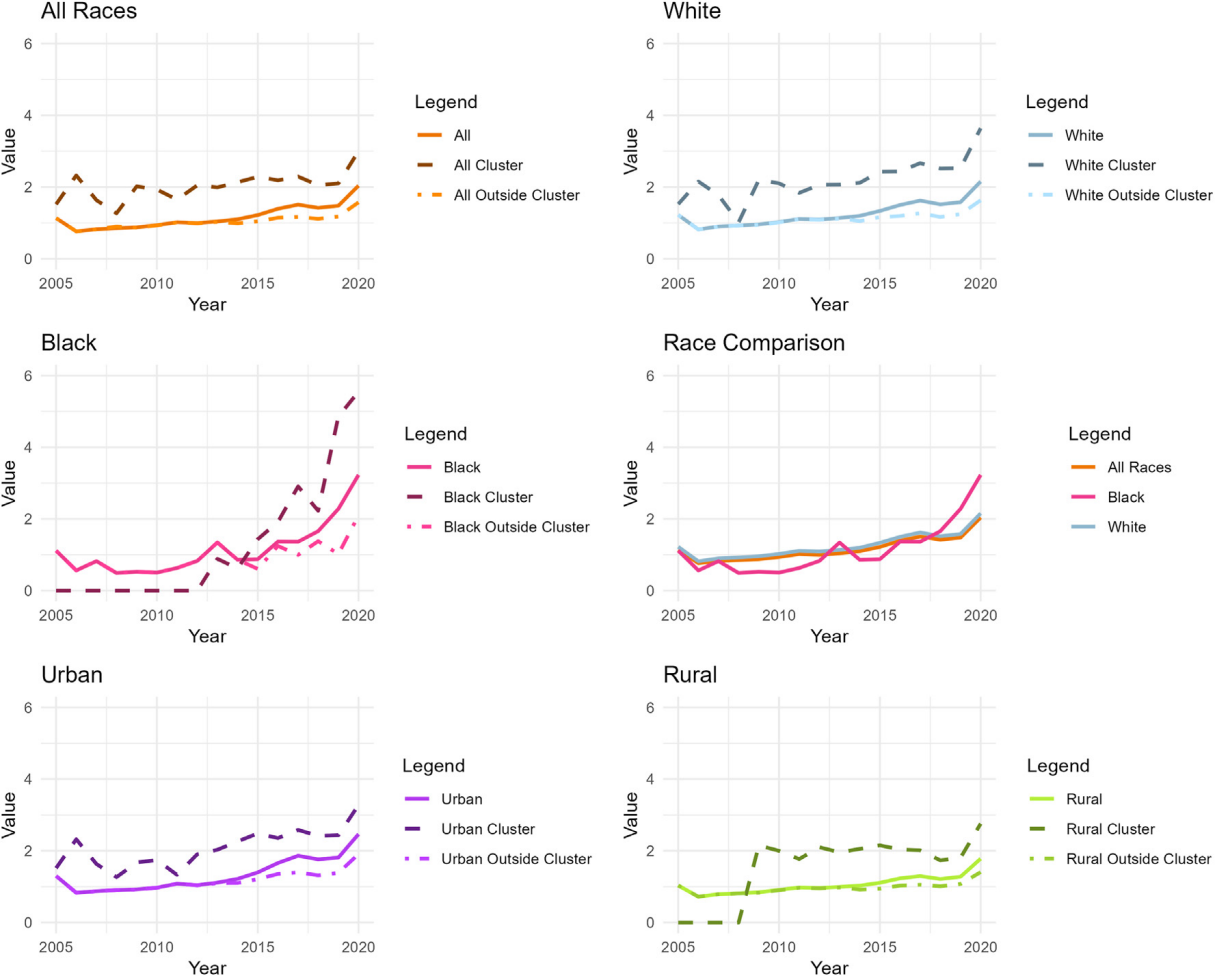
**Temporal trends in SUD mortality by subpopulation and clusters (2005–2020)***.* This figure presents the temporal trends in SUD-related mortality across different subpopulations in the contiguous United States from 2005 to 2020. Top panels, cumulative mortality graph for the general population (left), and comparative cumulative mortality across subpopulations (right). “Comparative cumulative mortality” refers to the accumulation of SUD-related deaths over time for specific subpopulations within the identified clusters, expressed as a comparison to the cumulative mortality observed outside of the clusters or the national average for the same subpopulation. This cumulative approach illustrates the differential impact of SUD mortality within clusters over time, showing how certain subpopulations or geographic settings (e.g., rural or urban areas) experience elevated mortality burdens relative to their counterparts. Middle panels, temporal trends for the White subpopulation (right), and temporal trends for the Black subpopulation (right). Bottom panels, temporal trends for the Urban subpopulation (left), and temporal trends for the rural population (right). These temporal graphs illustrate the evolving dynamics of the SUD epidemic, highlighting distinct temporal patterns and the emergence of clusters within different subpopulations over time.

Cumulative mortality graphs were produced for each subpopulation, contrasting the mortality for the subpopulation, for the counties within the SUD clusters in that subpopulation, and for the counties outside of these clusters in that subpopulation (Fig. 4). For the general population, there is a clear difference in the mortality observed inside of the clusters versus outside of the clusters, this contrast remains stable over time besides a sudden drop observed in 2008 for the mortality values inside of the clusters. Then, the estimated mortality observed outside of clusters drop below the estimated average mortality for the general population from 2012 onward.

The temporal trend of the SUD-related mortality in the White subpopulation closely resembles the patterns observed for the general population, with a reduction in the estimated SUD-related mortality observed in 2008 within the identified clusters. In contrast, the SUD-mortality in the Black subpopulation showed a different temporal pattern: a homogeneous spatial structure resulted in the absence of clusters until 2013, when we observe the first clusters rapidly emerge across the country. The emergence of these clusters is associated to an increase in SUD-related deaths in the Black subpopulation when compared with the national averages. Likewise, from 2016 onwards, the mortality rate observed in Black surpasses that of the White subpopulation. It became the highest SUD mortality rate among the observed subpopulations, significantly spiking in 2018 and onwards.

A distinct geographical disparity between urban and rural areas was observed in the mortality rates within and outside the identified clusters, exhibiting temporal fluctuations. Yet, urban counties predominantly characterized the clusters over the study period. Urban clusters were identified early in the study timeline, displaying variable mortality rates in relation to urban national averages. In contrast, clusters within rural areas emerged post-2009, consistently demonstrating a stable divergence in mortality rates when juxtaposed against the rural national average.

## Discussion

The spatiotemporal dynamics of the SUD epidemic in the US reveal the emergence of distinct localized micro-epidemics with unique characteristics. Using spatial scan statistics, clusters of elevated SUD mortality rates that exceeded expected values were identified. Initial clusters were detected in Washington (2005–2009) and Oklahoma (2009–2016). From 2013 to 2016, clusters proliferated in the western US, persisting until 2020, while clusters in the eastern US emerged from 2016 to 2018 and continued through the study period. Within the identified spatiotemporal clusters, the average mortality rate was 25.94 per 100,000 per person-years, compared to 13.61 per 100,000 per person-years in non-cluster regions, highlighting the need for region-specific public health interventions.

Socioeconomic patterns driving the emergence of these localized micro-epidemics are still under investigation. Studies suggest that different substances targeting specific demographics have initiated distinct epidemic phases.^4^^,^^9^^,^^23^ The initial phase involved prescription opioid misuse, followed by a surge in heroin-related deaths from 2007. The subsequent phase, characterized by fatalities from fentanyl and other synthetic opioids, marks a transition driven by heroin's increased availability and the challenges of obtaining prescription opioids. Recently, synthetic opioids misrepresented as other drugs have caused a spike in fatalities due to their potency heroin distribution in the US shows regional distinctions, with Colombian-sourced heroin prevalent in the East and Mexican “black tar” heroin in the West.^24^ Powdered heroin, common in the Northeast and Midwest, is more often adulterated with fentanyl than the solid “black tar” heroin in the West.^25^ These patterns illustrate the complex progression of the SUD epidemic, driven by varying supply and demand across different regions.

The spatiotemporal analysis highlights distinct regional patterns in SUD mortality across the United States, with clusters shifting from the West to the East over the study period. While the analysis did not pre-define regions such as the West Coast, Central region, and East Coast, the results naturally revealed distinct trends. Early clusters on the West Coast, particularly in Washington, were likely associated with the initial wave of prescription opioids and a subsequent increase in heroin availability. The Central region, including states like Oklahoma and Missouri, showed persistent clusters during the mid-study period, which may correspond to the transition from prescription opioids to heroin and the emergence of fentanyl. The East Coast, encompassing states like Ohio, Pennsylvania, and New Jersey, exhibited later clusters primarily associated with the rapid spread of synthetic opioids, particularly fentanyl.

These regional differences likely reflect variations in drug supply networks, state-level policies, healthcare availability, and socioeconomic conditions. Future analyses could benefit from explicitly dividing the country into these regional categories to further explore the unique characteristics and risk factors influencing the SUD epidemic in each area.

Our analysis revealed distinct SUD mortality patterns among racial subpopulations. Spatiotemporal scan statistics showed that clusters in the white subpopulation appeared early, similar to the general population pattern, while clusters in the black subpopulation emerged later, starting in 2013 and persisting through the study period. Within these clusters, the white subpopulation had a higher mortality rate (28.42 per 100,000 person-years) compared to non-cluster areas (14.83 per 100,000 person-years). For the black subpopulation, the rate within clusters was 33.16 per 100,000 person-years, versus 13.36 per 100,000 person-years outside. Notably, from 2016 onwards, the mortality rate among blacks surpassed that of whites, with a significant spike from 2018 onwards.

The early predominance of white mortality rates is linked to prescription opioid misuse, influenced by disparities in pain management access and systemic healthcare biases affecting black patients.^9^^,^^26^^,^^27^ The later emergence of clusters among blacks aligns with the rise of illicit synthetic opioids, particularly fentanyl, as shown by studies in St. Louis, Missouri, and Massachusetts, highlighting racial inequities in opioid overdose deaths disproportionately affecting the black population.^28^^,^^29^ These patterns emphasize the need for further research into the interplay between increased mortality, social determinants of health, and substance availability impacts on these demographic groups.

The estimated average SUD mortality rate during the study period was 1.30 per 10,000 individuals annually in urban areas, compared to 1.03 per 10,000 in rural areas. A segmented analysis between the eastern and western regions reveals a stark contrast: rural counties in the East had a lower likelihood of being classified as clusters compared to urban counties, while in the West, rural counties were more likely to form clusters than their urban counterparts. This analysis highlights a predominantly rural composition of clusters in the West.

This geographic disparity, with the West experiencing a predominantly rural SUD epidemic, can be attributed to several factors. Early cluster formations in the West, accelerated opioid overdose death rates in rural versus urban counties prior to 2010,^30^ and higher prescription opioid rates in these rural areas contribute to this trend.^31^^,^^32^ Rural-urban health disparities, compounded by the demographic and socioeconomic makeup of rural populations, characterized by a higher proportion of older adults with chronic pain and a significant non-Hispanic white demographic with greater access to opioid prescriptions,^33^^,^^34^ further explain the observed spatial distribution of SUD mortality. These findings underscore the complex interplay between geographic location, demographic characteristics, and access to healthcare resources in the unfolding of the SUD epidemic across the US.

Our research highlights that the national SUD epidemic is shaped by numerous localized micro-epidemics, necessitating targeted local interventions to mitigate the overall national burden. Precise and timely detection using fine spatial and temporal resolution is essential to identify populations at higher risk. These risks involve both demand-side factors, such as socioeconomic status, educational attainment, and employment rates, and supply-side dynamics, including the availability of specific substances. Demand-side factors are generally stable and spatially specific, while supply-side elements are more volatile, often shifting rapidly over time.^9^

The introduction of unregulated substances into the illicit market significantly impacts SUD mortality rates. The infiltration of fentanyl adulterated or substituted heroin (FASH) into the US market has markedly increased mortality, underscoring the critical role of supply dynamics in the epidemic's evolution.^35^ The use of spatiotemporal surveillance systems supports the creation of Early Warning Systems (EWS) for populations at increased risk due to demographic characteristics and exposure to new, unregulated substances. Such systems have been implemented at regional, national, and international levels, with entities like the United Nations Office of Drug and Crime (UNDOC), the Inter-American Drug Abuse Control Commission, and the European Monitoring Centre for Drugs and Drug Addiction providing foundational guidelines for EWS development and implementation.^36^

The National Drug Early Warning System (NDEWS) in the US specializes in disseminating hotspot alerts that identify deviations from expected drug-related incident rates within defined temporal windows. NDEWS, along with the State and National Overdose Web (SNOW) and the Florida Drug-Related Outcomes Surveillance and Tracking System (FROST), exemplifies the US's advanced surveillance efforts to monitor and respond to emerging drug threats.^37^^,^^38^ Additional early warning programs include DOSE,^39^ DAWN,^40^ and SUDOR.^41^ Despite these advancements, the spatial granularity of reported data often remains suboptimal for detailed risk assessments. Integrating broader datasets, including those from emergency departments, medical examiners, and hospital records, into existing surveillance frameworks can enhance the precision and utility of EWS for effectively addressing the SUD epidemic.

According to the CDC's Guiding Principles, addressing the SUD crisis requires innovative strategies to prevent overdoses and related harms.^42^ The SUD epidemic's dynamics are influenced by both demand, driven by socioeconomic factors within communities, and supply, dictated by the availability of controlled substances. Effective interventions must address these socioeconomic determinants and implement strict substance control policies. Additionally, recognizing individual decision-making in substance consumption suggests the need for comprehensive support, including educational initiatives, Naloxone distribution, and the development of surveillance and EWS.

EWS have proven beneficial for both policymakers and individuals directly affected by SUD. In Canada, provinces like British Columbia^39^ and Saskatchewan^43^ have implemented drug alert systems that provide real-time updates on hazardous substances within localities. These alerts offer detailed information about drugs, including their appearance and recent overdose locations, aiding informed decision-making among substance users. British Columbia also offers Drug Checking services, enabling individuals to test substances for dangerous adulterants like fentanyl and benzodiazepines. These services enhance community safety by providing accurate drug supply information. Although potentially contentious, such approaches emphasize the importance of engaging with communities affected by SUD to mitigate new risks. By analyzing drug testing information, a clearer understanding of the controlled substances market is gained, contrasting significantly with insights from clinical records. This facilitates targeted interventions at both individual and policy levels, illustrating a holistic approach to addressing the complex challenges of the SUD epidemic.

The identification of spatiotemporal clusters and the differential impact of the SUD epidemic on urban versus rural areas provides critical insights for designing and implementing effective EWS. Understanding the spatiotemporal dynamics and identifying high-risk regions and populations allows EWS to deliver targeted alerts and information, enabling rapid, focused responses. For example, recognizing that rural areas in the West are more likely to form clusters can guide regional strategies such as deploying mobile naloxone units or establishing localized drug checking services. Additionally, temporal analysis offers a framework for predicting potential future outbreaks, facilitating preemptive actions in emerging risk communities.

A detailed understanding of the dynamics of the epidemic supports innovative SUD management and prevention approaches. Policymakers can use our findings on demand and supply characteristics to craft multifaceted strategies addressing both immediate substance availability risks and deeper socioeconomic drivers of substance use. This might involve educational campaigns tailored to specific community needs and contexts, along with policies improving healthcare access and support services. Applying our results to EWS design and other interventions promises a more proactive, informed public health approach, mitigating the impact of the SUD epidemic.

At the local level, our findings highlight the importance of community-specific interventions. For instance, the identification of higher cluster formation in rural areas of the West suggests the need for enhanced resources and services in these regions. Local health departments should consider deploying mobile units for Naloxone distribution and establishing localized drug checking services to reduce overdose fatalities. Additionally, targeted educational campaigns that address the specific needs and contexts of rural populations can raise awareness about the risks of synthetic opioids like fentanyl.

On a national scale, our study supports the integration of advanced surveillance systems, such as FROST, SNOW, DOSE, SUDOR, and DAWN, to provide timely and precise alerts about specific substance threats. These Early Warning Systems can facilitate the rapid deployment of resources to areas identified as high-risk, allowing for a more proactive response to emerging trends in substance use and overdoses. Likewise, our findings on racial disparities in SUD mortality, particularly the late emergence of clusters among the black subpopulation, highlight the need for policies that address systemic biases within healthcare. National interventions should aim to improve access to culturally competent care and ensure equitable treatment for all demographic groups. This includes expanding access to medication-assisted treatment and other evidence-based therapies that have been shown to be effective in treating SUD.

Analyzing CDC mortality data is crucial for monitoring the SUD epidemic's evolving landscape. This comprehensive data analysis reveals patterns and trends essential for understanding the epidemic's complex dynamics. Future research should include such datasets to track changes in mortality rates, emerging SUD clusters, and demographic impact shifts. Ongoing analysis is vital for identifying new areas of concern, evaluating current intervention effectiveness, and adjusting strategies. As the SUD epidemic evolves, influenced by factors like new synthetic drugs and changing societal conditions, continuous monitoring of CDC mortality data will be invaluable for public health officials, researchers, and policymakers. This vigilance will enable a responsive, data-driven approach, guiding the development of targeted prevention and treatment strategies adaptable to the epidemic's complex dynamics. However, national policies should focus on standardizing substance classification across jurisdictions to ensure accurate and consistent data collection. This would enhance the quality of mortality data and enable more precise identification of spatiotemporal clusters. Furthermore, national strategies should promote the adoption of evidence-based practices in pain management and opioid prescribing to reduce disparities and prevent misuse.

This study has several limitations. First, reliance on ICD-10 codes for unintentional drug poisoning as a proxy for SUD mortality may introduce misclassification errors, as substances involved in deaths may be misidentified or unreported. This highlights the need for standardized substance classification across jurisdictions, particularly for emerging drugs like fentanyl. Second, the ecological and longitudinal nature of our analysis means findings reflect broader trends rather than individual–level associations. While spatial scan statistics effectively capture the spatiotemporal dynamics of SUD mortality, they do not account for substance-specific trends. Future research should explore substance-segregated analyses and assess the impact of local factors such as policy changes, trafficking routes, and healthcare access. Third, our spatiotemporal clustering approach was designed to detect clusters that emerge, persist, or disappear over time, capturing the epidemic's shifting geographic epicenter. The absence of persistent clusters suggests that external factors, including drug supply fluctuations, public health interventions, and regional reporting variability, may influence cluster formation. Further investigation is needed to assess how socioeconomic and healthcare disparities shape these patterns. Fourth, while we identified racial disparities in cluster formation, the study did not analyze the interaction between race and urban-rural status. Our findings indicate that rural clusters were more common among White populations, whereas the Black population experienced increasing urban clustering. Future studies should explore these intersections, incorporating socioeconomic and healthcare access data for a more comprehensive analysis. Fifth, RR estimates may be subject to bias due to cluster selection based on statistical significance. However, sensitivity analyses, varying Monte Carlo replications and excluding outliers, demonstrated consistent cluster stability, reinforcing the robustness of our findings. Finally, overdispersion remains a potential limitation, particularly in subgroup analyses. Higher misclassification rates in White and Black subpopulations suggest extra-Poisson variability, which the Poisson model in SaTScan may not fully account for. While our sensitivity analyses help mitigate this concern, future research should explore alternative statistical models, such as the negative binomial, to improve precision in heterogeneous populations.

This study reveals the shifting spatiotemporal dynamics of the SUD epidemic in the US, with the epidemic's epicenter transitioning from the western to the eastern US around 2016. We identified 27 significant clusters of elevated SUD mortality, persisting until 2020, with a notable rise in mortality among Black populations from 2013 onward. Rural areas in the West exhibited a higher propensity for clustering, while urban areas in the East became increasingly affected. These findings underscore the need for region-specific, adaptive public health interventions that account for evolving substance use patterns. In the West, sustaining harm reduction efforts such as Naloxone distribution and treatment access remains crucial, while in the East, interventions should focus on synthetic opioid surveillance, increased treatment availability, and harm reduction services. The disproportionate impact on Black communities highlights the urgency of addressing systemic healthcare disparities, ensuring equitable access to medication-assisted treatment (MAT), and strengthening culturally competent care. Real-time surveillance systems and continuous monitoring of SUD mortality trends are essential for early detection of emerging hotspots, guiding targeted interventions to mitigate the evolving crisis.

## Contributors

SE and DFC performed statistical analyses, data interpretation, and manuscript drafting. SE and DFC prepared figures and conducted data verification. All authors contributed to the study concept and design, data acquisition, and interpretation. All authors critically revised the manuscript for important intellectual content. SE and DFC had access to the raw data and verified the analyses. DFC had final responsibility for the decision to submit for publication. All authors read, reviewed, and approved the final manuscript.

**Access to data and data analysis**: Dr. Diego Cuadros and Santiago Escobar have full access to all the data in the study and takes responsibility for the integrity of the data and the accuracy of the data analysis.

## Data sharing statement

All data are available in public repositories: https://www.cdc.gov/nchs/nvss/nvss-restricted-data.htm.

## Declaration of interests

The authors have no conflicts of interest to declare.

## References

[1] Volkow N.D., Blanco C. (2023). Substance use disorders: a comprehensive update of classification, epidemiology, neurobiology, clinical aspects, treatment and prevention. World Psychiatry.

[2] CDC (2023). CDC national center for health statistics mortality data on CDC WONDER. https://wonder.cdc.gov/Deaths-by-Underlying-Cause.html.

[3] CDC (2019). NCHS data brief No. 343, July 2019. https://www.cdc.gov/nchs/products/databriefs/db343.htm.

[4] Hernández A.L.M., MacKinnon N.J., Branscum A.J., Cuadros D.F. (2021). "Know your epidemic, know your response": epidemiological assessment of the substance use disorder crisis in the United States. PLoS One.

[5] Ciccarone D. (2021). The rise of illicit fentanyls, stimulants and the fourth wave of the opioid overdose crisis. Curr Opin Psychiatry.

[6] Cuadros D.F.B.A., Moreno C.M., MacKinnon N.J. (2023). Narrative minireview of the spatial epidemiology of substance use disorder in the United States: who is at risk and where?. World J Clin Cases.

[7] Sun F. (2022). Rurality and opioid prescribing rates in U.S. counties from 2006 to 2018: a spatiotemporal investigation. Soc Sci Med.

[8] Post L.A., Lundberg A., Moss C.B. (2022). Geographic trends in opioid overdoses in the US from 1999 to 2020. JAMA Netw Open.

[9] D. C (2019). The triple wave epidemic: supply and demand drivers of the US opioid overdose crisis. Int J Drug Policy.

[10] CDC (2022). Restricted-use vital statistics data. https://www.cdc.gov/nchs/nvss/nvss-restricted-data.htm.

[11] World Health Organization (2019). International classification of diseases for mortality and morbidity statistics (10th Revision). https://www.who.int/standards/classifications/classification-of-diseases.

[12] Bureau U.C. Population and housing units estimates. https://www.census.gov/programs-surveys/popest.html.

[13] Ingram D.D., Franco S. (2014). 2013 NCHS urban–rural classification scheme for counties. Vital Health Stat.

[14] M. K. (1997). A spatial scan statistic. Commun Stat Theor Methods.

[15] Wand H., Ramjee G. (2010). Targeting the hotspots: investigating spatial and demographic variations in HIV infection in small communities in South Africa. J Int AIDS Soc.

[16] Ryan J., Mbui J., Rashid J. (2006). Spatial clustering and epidemiological aspects of visceral Leishmaniasis in two endemic villages, Baringo District, Kenya. Am J Trop Med Hyg.

[17] Kulldorf M., Song C., Gregoria D., Samociuk H., DeChello L. (2006). Cancer map patterns: are they random or not?. Am J Prev Med.

[18] Cuadros D., Awad S., Abu-Raddad L. (2013). Mapping HIV clustering: a strategy for identifying populations at high risk of HIV infection in sub-Saharan Africa. Int J Health Geogr.

[19] Jones P., Gunnell D., Platt S. (2013). Identifying probable suicide clusters in Wales using national mortality data. PLoS One.

[20] Malleson N., Andresen M.A. (2015). Spatio-temporal crime hotspots and the ambient population. Crime Sci.

[21] Ruiz-Grosso P., Miranda J.J., Gilman R.H. (2016). Spatial distribution of individuals with symptoms of depression in a periurban area in Lima: an example from Peru. Ann Epidemiol.

[22] Wilkinson L. (2011).

[23] Hernandez A.B.A., Li J., MacKinnon N.J., Hincapie A.L., Cuadros D.F. (2020). Epidemiological and geospatial profile of the prescription opioid crisis in Ohio, United States. Sci Rep.

[24] Ciccarone D. (2009). Heroin in brown, black and white: structural factors and medical consequences in the US heroin market. Int J Drug Pol.

[25] Carroll J.J., Marshall B.D.L., Rich J.D., Green T.C. (2017). Exposure to fentanylcontaminated heroin and overdose risk among illicit opioid users in Rhode Island: a mixed methods study. Int J Drug Pol.

[26] Schuler M.S.S.T., Wong E.C., Wong E.C. (2021). Racial/ethnic differences in prescription opioid misuse and heroin use among a national sample, 1999-2018. Drug Alcohol Depend.

[27] Meghani S.H.B.E., Gallagher R.M. (2012). Time to take stock: a meta-analysis and systematic review of analgesic treatment disparities for pain in the United States. Pain Med.

[28] Banks D.E.S.S., Paschke M.E., Shacham E., Nance M., Cavazos-Rehg P., Winograd R.P. (2023). Examining increasing racial inequities in opioid overdose deaths: a spatiotemporal analysis of black and white decedents in St. Louis, Missouri, 2011-2021. J Urban Health.

[29] Stopka T.J.L.M., Li X., Bernson D. (2023). Opioid-related mortality: dynamic temporal and spatial trends by drug type and demographic subpopulations, Massachusetts, 2005-2021. Drug Alcohol Depend.

[30] Monnat S.M. (2022). Demographic and geographic variation in fatal drug overdoses in the United States, 1999–2020. Ann Am Acad Pol Soc Sci.

[31] Guy G.P., Zhang K., Bohm M.K. (2017).

[32] García M.C., Heilig C.M., Lee S.H. (2019). Opioid prescribing rates in nonmetropolitan and metropolitan counties among primary care providers using an electronic health record system — United States, 2014–2017. MMWR Morb Mortal Wkly Rep.

[33] Verheij R.A. (1996). Explaining urban-rural variations in health: a review of interactions between individual and environment. Soc Sci Med.

[34] Katherine M., Keyes M.C., Joanne E B., Jennifer R H., Sandro G. (2014). Understanding the rural–urban differences in nonmedical prescription opioid use and abuse in the United States. Am J Publ Health.

[35] Mars S.G.R.D., Ciccarone D. (2019). Illicit fentanyls in the opioid street market: desired or imposed?. Addiction.

[36] UNDOC Early warning system on new psychoactive substances and emerging drug phenomena implementation manual. https://www.unodc.org/LSS/Home/NPS.

[37] NDEWS (2020). National drug early warning system. https://ndews.org/about/.

[38] NDEWS Florida drug-related outcomes surveillance and tracking system web (SNOW). https://frost.med.ufl.edu/.

[39] BCCDC British Columbia center's for disease control toxic and drug health alerts system. https://towardtheheart.com/alerts.

[40] SAMHSA Drug abuse warning network (DAWN). https://www.samhsa.gov/data/data-we-collect/dawn-drug-abuse-warning-network.

[41] CDC State unintentional drug overdose reporting system (SUDORS). https://www.cdc.gov/drugoverdose/fatal/dashboard/index.html.

[42] CDC Drug overdose prevention guiding principles. https://www.cdc.gov/drugoverdose/prevention/index.html.

[43] Health SMo Saskatchewan's ministry of health drug alert system. https://www.saskatchewan.ca/drug-alerts.

