# Supplementary material for: Spatiotemporal analysis of substance use disorder mortality in the United States: an observational study of emerging hotspots and vulnerable populations (2005–2020)
Source: Lancet Reg Health Am. 2025 Apr 4;45:101075. doi: 10.1016/j.lana.2025.101075 (PMC12003021; doi:10.1016/j.lana.2025.101075)
Supplement: Supplementary Materials [file mmc1.pdf]

## Supplementary Materials for:

# Spatiotemporal Analysis of Substance Use Disorder Mortality in the United States: An Observational study of Emerging Hotspots and Vulnerable Populations (2005–2020)

Santiago Escobar, Neil J. MacKinnon, Preshit Ambade, Zach Hoffman, Diego F. Cuadros

## Table of contents

| | Page |
|---|---|
| ***1. Supplementary Methods*** | 1 |
| *1.1. Spatiotemporal clustering analysis* | 1 |
| *1.2. Confidence interval estimations* | 3 |
| *1.3. Sensitivity analysis* | 6 |
| ***2. Supplementary results*** | 7 |
| *2.1. General estimations* | 7 |
| *2.2. SaTScan results* | 10 |
| *2.3. Sensitivity analysis* | 17 |
| ***3. Supplementary Limitations of the study*** | 28 |
| ***4. References*** | 30 |

## 1. SUPPLEMTARY METHODS

### *1.1 Spatiotemporal clustering analysis*

A county-level spatiotemporal clustering analysis was conducted to identify geographical clusters of high numbers of SUD-related deaths that persist for at least two or more years using Kulldorff's spatial scan statistics[1] implemented in the SaTScan software. The analysis was performed for the general population, as well as for the White and Black subpopulations. Scan statistics are widely used for cluster detection in epidemiology [2-5], social sciences,[6] crime mapping,[7] and, very recently, in mental health,[8] among other applications. A detailed description of the spatial scan statistics is provided elsewhere.[1,4]

The space-time scan statistic with a Poisson model is a validated and widely applied method in epidemiological research for cluster detection across various health domains. The Poisson model in SaTScan has been extensively utilized in studies of SUD and opioid mortality,[9-13] cancer surveillance,[4,14,15] infectious disease outbreaks,[16] and mental health outcomes,[17-19] demonstrating its adaptability to diverse public health applications. Given the large sample size of our study, its extended temporal coverage (2005–2020), and the broad geographic scope of the identified clusters, the Poisson model in SaTScan is appropriate for capturing SUD mortality patterns on a national scale. Briefly, Kulldorff's scan statistics are used to detect high-risk spatiotemporal clusters of cases (i.e. SUD-related deaths) by gradually scanning a cylindrical window, with a base corresponding to space and the height corresponding to time. Cylinders of varying radii spanned the study region and time-period to identify areas where SUD-related deaths were clustered. The cylinder varied continuously in both location and radius, thus creating and testing a very large number of distinct potential clusters. A log-likelihood ratio of the test scan statistic is constructed based on the actual number of occurrences and the expected number of occurrences inside and outside the cylinder. The spatial scan statistic identifies clusters based on statistical significance ($p < 0.05$) using Monte Carlo simulations (using the default value of 999 iterations); however, the analysis extends beyond significance testing to provide estimation of the relative risk (RR) and mortality rates within and outside the identified clusters. This approach ensures that the magnitude and intensity of the clusters are quantified, regardless of statistical significance, allowing for a more comprehensive assessment of the SUD mortality patterns across different regions.

We applied a Poisson likelihood function, which is suitable for count data, to model the occurrence of SUD mortality events in relation to the population at risk within each county. This approach enables the identification of clusters with significantly elevated risk while accounting for the underlying population distribution. The null hypothesis for the spatiotemporal analysis posits that the risk of SUD mortality is uniformly distributed across all counties and time periods, in proportion to the population at risk within each spatial unit. Under this null hypothesis, the expected number of SUD-related deaths in each county is calculated based on the population size and overall mortality rate. The spatial scan statistic compares the observed number of cases within each potential cluster against this expected value, identifying statistically significant clusters where the observed cases exceed the expected count under the null hypothesis.

In the space-time scan statistic using the Poisson model, SaTScan calculates RR for each identified cylindrical cluster using the formula:

$$RR = \frac{c}{e}$$

where $c$ is the observed number of cases within the space-time cylinder, and $e$ is the expected number of cases within that cylinder.

The expected cases $e(A)$ for a given cylinder $A$ are calculated as follows:

$$e(A) = \mu(A) \times \frac{C}{\mu(G)}$$

where $\mu(A)$ represents the population-years within the space-time cylinder $A$, $C$ is the total number of cases in the entire study period, and $\mu(G)$ is the total population-years in the entire study region. This method inherently accounts for both spatial and temporal dimensions, as the population-years metric incorporates the size of the population in the geographic area and the temporal duration of the cluster.[1]

This calculation method ensures that the RR accurately reflects the elevated risk by accounting for the population at risk and the time period of the cluster, providing a robust measure of the spatiotemporal distribution of SUD mortality. By using Kulldorff's spatial scan statistic in SaTScan with a Poisson model, the analysis leverages the entire population dataset, enhancing the precision and reliability of the identified clusters. The county-level granularity provides sufficient population size per spatial unit, supporting the detection of statistically significant clusters across diverse regions and subpopulations. Lastly, to account for variability and enhance robustness in relative risk estimates, we employed a bootstrapping method to calculate confidence intervals around RR values (see Supplementary Materials for details on methodology and sample size selection).

### *1.2. Confidence Interval Estimations*

Since SaTScan does not directly generate confidence interval (CI) estimations, to quantify the uncertainty associated with the relative risks (RRs) of spatiotemporal clusters identified in our analysis, we employed a bootstrapping method. In our analysis, we selected bootstrapping as the primary method for estimating CIs over traditional non-parametric approaches due to its flexibility and robustness, especially in contexts involving count data with variability, such as substance use disorder (SUD) mortality rates. Bootstrapping is particularly well-suited for mortality data where the distribution of events may not conform neatly to parametric assumptions. This approach resamples the observed data with replacement, generating a distribution of estimates that reflects the true variability inherent in our dataset, without assuming a specific distribution shape. This is essential when working with spatial scan statistics in epidemiology, as SUD mortality often exhibits substantial geographic and temporal variability that could lead to underestimated uncertainty in CIs if traditional parametric methods were used. Additionally, bootstrapping aligns well with the Poisson model used in our primary analysis. In spatial scan statistics, the Poisson distribution is employed to model the count of events within

clusters. This assumption is directly compatible with bootstrapping since each bootstrap sample maintains the underlying count structure without altering the expected rate of events in a way that would conflict with the Poisson assumption. The use of bootstrapping, therefore, reinforces the robustness of our CI estimates, as it respects the intrinsic variability of the observed counts and maintains the integrity of the Poisson distribution assumptions.

We first identified the observed and expected number of cases within each cluster identified in SaTScan over its duration (i.e., start year to end year). The observed counts were based on mortality data, while the expected counts were derived from population data and adjusted using national averages. To account for the variability inherent in the observed data, we simulated 1,000 bootstrap samples for each cluster using a Poisson distribution with a mean equal to the observed number of cases ($\lambda$ = observed cases). We chose 1,000 iterations for our bootstrap sample size to balance computational efficiency with the reliability of the CI estimates. In epidemiological research, this number of iterations is often considered sufficient for stabilizing the CI estimates and capturing the underlying variability within the data. Increasing the number of iterations further would yield diminishing returns in terms of precision while significantly increasing computational demands. Previous studies utilizing spatial scan statistics have found 1,000 iterations adequate for robust CI estimates, providing a stable distribution of results without excessive computational cost. Therefore, selecting 1,000 iterations for our bootstrap process ensures a reliable estimation of CIs that is both efficient and statistically sound.

For each bootstrap sample, we calculated the RR as the ratio of the bootstrap case count (adjusted for the time length of the cluster) to the expected case count (also adjusted for the time length), where "bootstrap cases" refers to the simulated count from the Poisson distribution, and *time length* is the duration of the cluster in years. This normalization ensured that the RR accounts for the duration over which the cluster was observed, making it comparable across clusters of different durations.

To estimate the 95% CI for each cluster's RR, we applied the percentile method using the distribution of the 1,000 bootstrap RRs. The lower bound of the CI was determined as the 2.5th percentile of the bootstrap RRs, and the upper bound as the 97.5th percentile. This non-parametric approach is suitable as it does not assume symmetry or normality in the RR distribution, making it robust for epidemiological data that might not follow these properties. The resulting CI provides an interval within which the true RR is likely to fall with 95% confidence, capturing the uncertainty associated with each cluster's RR.

Additionally, to ensure that the bootstrap distribution of the RRs accurately represented the variability of the observed data, we visually inspected histograms of the bootstrap RRs for select clusters (Supplementary Figure 1). This step allowed us to verify that the distribution was appropriately centered around the observed RR and that the percentile-based CI was well-aligned with the spread of the bootstrap data. By employing this bootstrapping approach, we obtained robust estimates of the CIs for the RRs associated with each cluster, providing a comprehensive understanding of the uncertainty and variability inherent in the identified clusters of elevated SUD mortality identified using SaTScan.

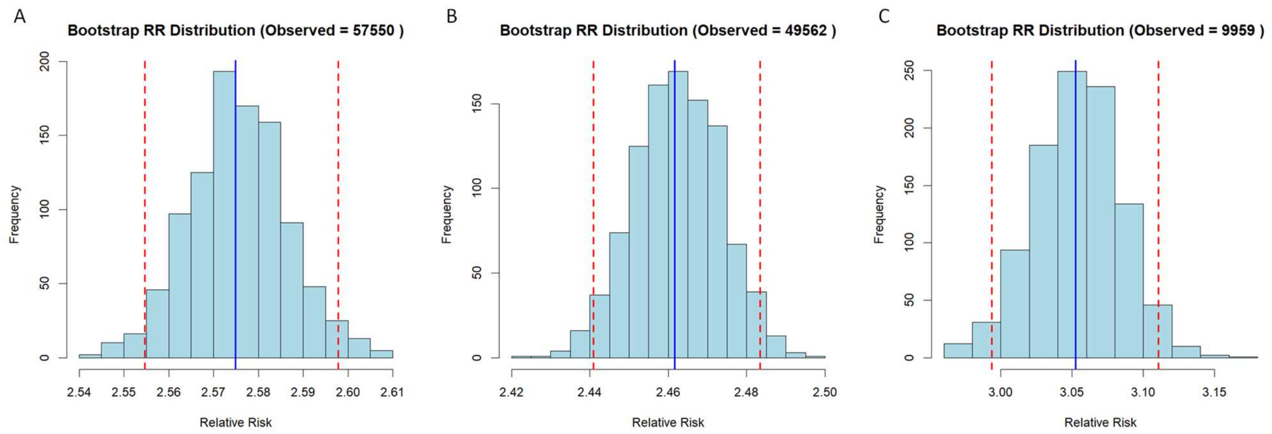


***Supplementary Figure 1***. Histograms of the bootstrap RRs for select clusters for A) total population, B) White population, and C) Black population.

### *1.3. Sensitivity Analysis*

To assess the robustness of our findings, we conducted two complementary sensitivity analyses: (1) varying the number of Monte Carlo replications and (2) conducting an outlier exclusion analysis. Each of these analyses serves a distinct purpose in validating the stability and reliability of our results under different conditions.

#### 1.3.1. Monte Carlo Simulation Sensitivity Analysis

The first sensitivity analysis involved varying the number of Monte Carlo replications used to assess statistical significance. In the main analysis, we employed the standard 999 replications as recommended by SaTScan, then increased the replications to intermediate (5,000) and maximum (9,999) levels to examine whether changes in the number of iterations impacted the stability of identified clusters. Monte Carlo replications are critical in spatial scan statistics as they determine the p-values for each potential cluster, allowing us to assess statistical significance robustly. This analysis validates the robustness of our findings by demonstrating that identified clusters are consistent across different replication levels. Stability across varying replications indicates that the observed clusters are not products of random variation introduced by the Monte Carlo procedure. Thus, this analysis provides confidence that our cluster identification is reliable and not significantly influenced by the number of replications selected, reinforcing the validity of our findings under different simulation conditions.

#### 1.3.2. Outlier Exclusion Analysis

The second sensitivity analysis focused on outlier exclusion, specifically by removing counties within the 98th percentile of SUD-related death counts. This threshold was selected to exclude extreme values without unnecessarily reducing the dataset. High-count outliers may exert undue influence on cluster detection and RR estimates, potentially inflating RR values if these clusters are heavily weighted by a few counties with disproportionately high mortality rates. By rerunning the analysis on this truncated dataset, we were able to assess the resilience of our clusters in the absence of high-count outliers. Consistency in results between the full dataset and the truncated dataset indicates that our findings are robust and not overly dependent on extreme cases, suggesting that the identified clusters reflect underlying patterns in SUD mortality across broader population groups rather than isolated high-mortality areas. This test provides additional generalizability to our results, as it confirms that RR estimates are not artificially elevated by outlier values and are representative of the larger dataset.

Together, these two sensitivity analyses provide a comprehensive validation of our results, demonstrating that the identified clusters and RR estimates are stable across different simulation parameters and are not unduly influenced by extreme values within the data.

## 2. SUPPLEMENTARY RESULTS

### *2.1. General Estimations*

Supplementary Figure 2 illustrates the trends in SUD-related death rates per 100,000 individuals from 2005 to 2020, showing a steady increase across the total, White, and Black populations. While the total and White populations follow a similar trajectory with gradual increases until 2014 and a steeper rise thereafter, the Black population exhibits a distinctive pattern. Beginning around 2015, death rates in the Black population escalate sharply, surpassing those of the total and White populations by 2020, reaching over 35 deaths per 100,000. This rapid, late increase in mortality among Black individuals highlights a recent intensification of the SUD crisis in this group, underscoring the urgency for targeted interventions to address growing disparities in SUD-related outcomes.

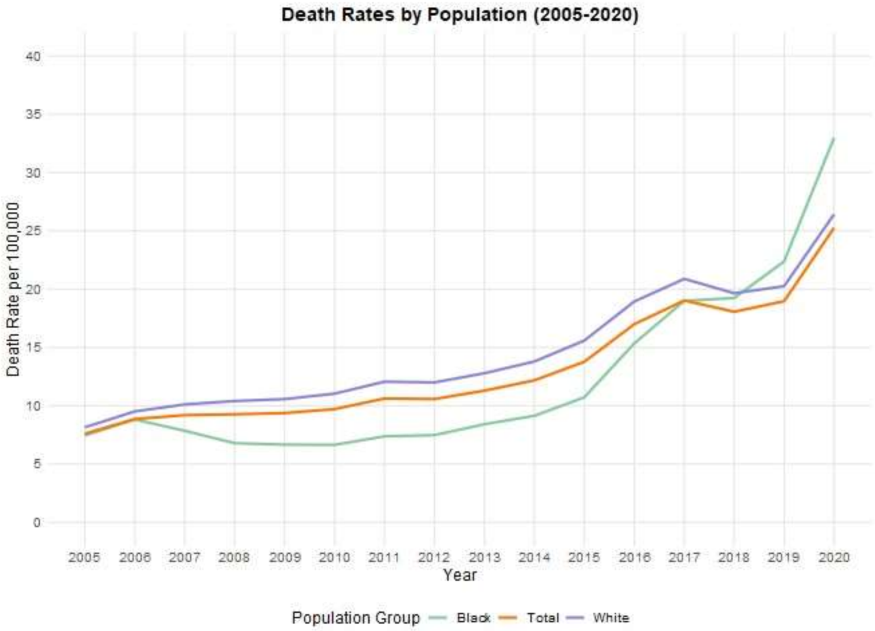


***Supplementary Figure 2.*** Temporal trend of the mortality rate for the total (orange line), Black (green line) and White (purple line) populations from 2005 to 2020.

***Supplementary Table 1***. General estimations for the total population and the Black and White populations included.

| Year | Total Population | | | Black Population | | | White Population | | |
|---|---|---|---|---|---|---|---|---|---|
| | ***Total Deaths*** | ***Population*** | ***Death Rate (95% CI)*** | ***Total Deaths*** | ***Population*** | ***Death Rate (95% CI)*** | ***Total Deaths*** | ***Population*** | ***Death Rate (95% CI)*** |
| **2005** | 22,310 | 293,578,002 | 7.60 (7.50 - 7.70) | 2,825 | 37,911,629 | 7.45 (7.18 - 7.73) | 19,121 | 234,656,286 | 8.15 (8.03 - 8.26) |
| **2006** | 26,270 | 296,388,732 | 8.86 (8.76 - 8.97) | 3,386 | 38,393,416 | 8.82 (8.52 - 9.12) | 22,470 | 236,136,877 | 9.52 (9.39 - 9.64) |
| **2007** | 27,506 | 299,229,063 | 9.19 (9.08 - 9.30) | 3,052 | 38,887,117 | 7.85 (7.57 - 8.13) | 24,025 | 237,561,464 | 10.11 (9.99 - 10.24) |
| **2008** | 27,991 | 302,068,148 | 9.27 (9.16 - 9.38) | 2,673 | 39,380,995 | 6.79 (6.53 - 7.04) | 24,860 | 238,959,779 | 10.40 (10.27 - 10.53) |
| **2009** | 28,559 | 304,719,743 | 9.37 (9.26 - 9.48) | 2,656 | 39,854,028 | 6.66 (6.41 - 6.92) | 25,388 | 240,205,178 | 10.57 (10.44 - 10.70) |
| **2010** | 29,835 | 307,265,840 | 9.71 (9.60 - 9.82) | 2,677 | 40,309,013 | 6.64 (6.39 - 6.89) | 26,656 | 241,455,959 | 11.04 (10.91 - 11.17) |
| **2011** | 32,864 | 309,455,417 | 10.62 (10.51 - 10.73) | 3,006 | 40,733,370 | 7.38 (7.12 - 7.64) | 29,252 | 242,449,374 | 12.07 (11.93 - 12.20) |
| **2012** | 32,966 | 311,705,743 | 10.58 (10.46 - 10.69) | 3,078 | 41,172,324 | 7.48 (7.21 - 7.74) | 29,222 | 243,503,298 | 12.00 (11.86 - 12.14) |
| **2013** | 35,453 | 313,848,404 | 11.30 (11.18 - 11.41) | 3,501 | 41,597,045 | 8.42 (8.14 - 8.70) | 31,268 | 244,449,052 | 12.79 (12.65 - 12.93) |
| **2014** | 38,503 | 316,150,187 | 12.18 (12.06 - 12.30) | 3,840 | 42,034,546 | 9.14 (8.85 - 9.42) | 33,873 | 245,490,857 | 13.80 (13.65 - 13.95) |
| **2015** | 43,881 | 318,475,613 | 13.78 (13.65 - 13.91) | 4,557 | 42,475,720 | 10.73 (10.42 - 11.04) | 38,440 | 246,521,205 | 15.59 (15.44 - 15.75) |
| **2016** | 54,523 | 320,772,296 | 17.00 (16.85 - 17.14) | 6,585 | 42,911,888 | 15.35 (14.97 - 15.72) | 46,890 | 247,550,645 | 18.94 (18.77 - 19.11) |
| **2017** | 61,029 | 320,772,296 | 19.03 (18.87 - 19.18) | 8,152 | 42,911,888 | 19.00 (18.58 - 19.41) | 51,692 | 247,550,645 | 20.88 (20.70 - 21.06) |
| **2018** | 58,622 | 324,531,769 | 18.06 (17.92 - 18.21) | 8,403 | 43,673,243 | 19.24 (18.83 - 19.65) | 48,958 | 249,114,216 | 19.65 (19.48 - 19.83) |
| **2019** | 61,859 | 326,092,106 | 18.97 (18.82 - 19.12) | 9,851 | 44,017,020 | 22.38 (21.94 - 22.82) | 50,572 | 249,683,385 | 20.25 (20.08 - 20.43) |
| **2020** | 83,173 | 329,220,214 | 25.26 (25.09 - 25.44) | 14,785 | 44,820,002 | 32.99 (32.46 - 33.52) | 66,283 | 250,844,042 | 26.42 (26.22 - 26.63) |

Supplementary Table 1 summarizes the general estimations of the total population and the Black and White populations included in the study with their corresponding number of SUD related deaths and the mortality rate per year.

### *2.2. SaTScan Results*

Supplementary Tables 2 to 5 summarizes the results from the SaTScan analyses for the three studied populations. Supplementary Figure 3 illustrates the RR and length duration of each of the clusters identified for each population. For the total population (Panel A) and White population (Panel B), clusters in both populations appear consistently across the study period, with RRs mostly stable and slightly elevated above the baseline. This pattern suggests that SUD mortality clusters for these populations are relatively steady over time. For the Black population (Panel C), this population shows a notable emergence of clusters later in the study period, primarily from 2014 onward. These later clusters exhibit higher RRs, some exceeding 5, and larger confidence intervals, indicating both an increase in relative risk and variability in mortality risk for Black communities in recent years. This late emergence pattern highlights a concerning, concentrated rise in SUD mortality among the Black population in specific recent time periods, pointing to potential factors or disparities that may have intensified SUD impacts in these communities.

***Supplementary Table 2***. SaTScan results for the total population, including the time period of the cluster, the number of SUD-related deaths observed, the number of SUD-related deaths expected, the total population within the cluster, the estimated relative risk (RR) with their corresponding 95% confidence interval (CI), the mortality rate inside and outside the identified cluster with their corresponding 95% CI.

| Cluster | Period | Observed | Expected | Population | P Value | RR (95% CI) | Inside Rate (95% CI) | Outside Rate (95% CI) |
|---|---|---|---|---|---|---|---|---|
| 1 | 2014-2020 | 57,550 | 22,350 | 23,811,336 | <0.0001 | 2.57 (2.55-2.59) | 34.53 (33.78-35.27) | 13.41 (12.94-13.87) |
| 2 | 2016-2020 | 61,581 | 31,536 | 46,544,917 | <0.0001 | 1.95 (1.93-1.96) | 26.46 (25.99-26.93) | 13.55 (13.22-13.89) |
| 3 | 2016-2020 | 21,662 | 11,099 | 15,387,781 | <0.0001 | 1.95 (1.92-1.97) | 28.15 (27.32-28.99) | 14.43 (13.83-15.03) |
| 4 | 2016-2020 | 23,137 | 14,182 | 21,118,655 | <0.0001 | 1.63 (1.61-1.65) | 21.91 (21.28-22.54) | 13.43 (12.94-13.93) |
| 5 | 2015-2020 | 14,152 | 8,200 | 9,792,386 | <0.0001 | 1.72 (1.69-1.75) | 24.09 (23.11-25.06) | 13.96 (13.22-14.70) |
| 6 | 2017-2020 | 15,552 | 9,351 | 16,602,847 | <0.0001 | 1.66 (1.63-1.68) | 23.42 (22.68-24.15) | 14.08 (13.51-14.65) |
| 7 | 2013-2020 | 5,922 | 2,642 | 2,407,917 | <0.0001 | 2.24 (2.19-2.30) | 30.74 (28.53-32.96) | 13.72 (12.24-15.19) |
| 8 | 2017-2020 | 7,873 | 4,528 | 7,869,812 | <0.0001 | 1.73 (1.69-1.77) | 20.01 (19.02-21.00) | 11.51 (10.76-12.26) |
| 9 | 2014-2020 | 4,076 | 2,098 | 2,172,664 | <0.0001 | 1.94 (1.88-2.00) | 26.80 (24.62-28.98) | 13.79 (12.23-15.36) |
| 10 | 2015-2020 | 2,210 | 1,186 | 1,476,346 | <0.0001 | 1.86 (1.79-1.94) | 24.95 (22.40-27.50) | 13.39 (11.52-15.26) |
| 11 | 2013-2020 | 1,705 | 923 | 862,011 | <0.0001 | 1.84 (1.75-1.93) | 24.72 (21.40-28.04) | 13.38 (10.94-15.83) |
| 12 | 2016-2020 | 1,497 | 817 | 1,206,801 | <0.0001 | 1.83 (1.73-1.92) | 24.81 (22.00-27.62) | 13.54 (11.46-15.62) |
| 13 | 2013-2020 | 3,492 | 2,487 | 2,203,970 | <0.0001 | 1.40 (1.35-1.45) | 19.81 (17.95-21.66) | 14.11 (12.54-15.67) |
| 14 | 2009-2016 | 3,998 | 2,941 | 2,743,513 | <0.0001 | 1.35 (1.31-1.40) | 18.22 (16.62-19.81) | 13.40 (12.03-14.77) |
| 15 | 2017-2020 | 4,221 | 3,179 | 5,904,435 | <0.0001 | 1.32 (1.28-1.36) | 17.87 (16.79-18.95) | 13.46 (12.52-14.40) |
| 16 | 2018-2020 | 3,701 | 2,817 | 6,463,134 | <0.0001 | 1.31 (1.27-1.35) | 19.09 (18.02-20.15) | 14.53 (13.60-15.46) |
| 17 | 2018-2020 | 836 | 469 | 1,101,808 | <0.0001 | 1.78 (1.66-1.89) | 25.29 (22.32-28.26) | 14.19 (11.96-16.41) |
| 18 | 2014-2020 | 1,857 | 1,408 | 1,420,172 | <0.0001 | 1.31 (1.25-1.38) | 18.68 (16.43-20.93) | 14.16 (12.21-16.12) |
| 19 | 2014-2020 | 590 | 367 | 383,315 | <0.0001 | 1.60 (1.47-1.74) | 21.99 (17.29-26.68) | 13.68 (9.98-17.38) |
| 20 | 2017-2020 | 1,245 | 964 | 1,715,171 | <0.0001 | 1.29 (1.22-1.35) | 18.15 (16.13-20.16) | 14.05 (12.28-15.83) |
| 21 | 2018-2020 | 1,368 | 1,086 | 2,478,720 | <0.0001 | 1.26 (1.19-1.32) | 18.40 (16.71-20.09) | 14.60 (13.10-16.11) |

| Cluster | Period | Observed | Expected | Population | P Value | RR (95% CI) | Inside Rate (95% CI) | Outside Rate (95% CI) |
|---|---|---|---|---|---|---|---|---|
| 22 | 2005-2008 | 399 | 274 | 546,567 | <0.0001 | 1.45 (1.32-1.59) | 18.25 (14.67-21.83) | 12.53 (9.56-15.50) |
| 23 | 2014-2020 | 597 | 446 | 474,316 | <0.0001 | 1.33 (1.24-1.44) | 17.98 (14.16-21.80) | 13.43 (10.13-16.73) |
| 24 | 2017-2020 | 527 | 386 | 705,987 | <0.0001 | 1.36 (1.24-1.48) | 18.66 (15.48-21.85) | 13.67 (10.94-16.40) |
| 25 | 2018-2020 | 349 | 242 | 592,023 | <0.0001 | 1.44 (1.28-1.59) | 19.65 (16.08-23.22) | 13.63 (10.65-16.60) |
| 26 | 2017-2020 | 175 | 104 | 187,158 | <0.0001 | 1.68 (1.43-1.94) | 23.38 (16.45-30.30) | 13.89 (8.55-19.23) |
| 27 | 2016-2020 | 80 | 39 | 60,114 | 0.012 | 2.05 (1.59-2.54) | 26.62 (13.57-39.66) | 12.98 (3.87-22.08) |

***Supplementary Table 3***. SaTScan results for the White population, including the time period of the cluster, the number of SUD-related deaths observed, the number of SUD-related deaths expected, the total population within the cluster, the estimated relative risk (RR) with their corresponding 95% confidence interval (CI), the mortality rate inside and outside the identified cluster with their corresponding 95% CI.

| Cluster | Period | Observed | Expected | Population | P Value | RR (95% CI) | Inside Rate (95% CI) | Outside Rate (95% CI) |
|---|---|---|---|---|---|---|---|---|
| 1 | 2014-2020 | 49,562 | 20,134 | 19,751,330 | <0.0001 | 2.46 (2.44-2.48) | 35.85 (35.01-36.68) | 14.56 (14.03-15.09) |
| 2 | 2016-2020 | 51,193 | 25,513 | 35,090,639 | <0.0001 | 2.01 (1.99-2.02) | 29.18 (28.61-29.74) | 14.54 (14.14-14.94) |
| 3 | 2016-2020 | 19,566 | 9,416 | 12,096,506 | <0.0001 | 2.07 (2.04-2.10) | 32.35 (31.34-33.36) | 15.57 (14.86-16.27) |
| 4 | 2016-2020 | 14,399 | 8,402 | 10,994,563 | <0.0001 | 1.71 (1.68-1.74) | 26.19 (25.24-27.15) | 15.28 (14.55-16.01) |
| 5 | 2014-2020 | 14,085 | 8,443 | 8,019,692 | <0.0001 | 1.66 (1.64-1.69) | 25.09 (23.99-26.19) | 15.04 (14.19-15.89) |
| 6 | 2013-2020 | 4,492 | 1,900 | 1,599,421 | <0.0001 | 2.36 (2.30-2.43) | 35.11 (32.20-38.01) | 14.85 (12.96-16.74) |
| 7 | 2016-2020 | 3,513 | 1,646 | 2,255,696 | <0.0001 | 2.13 (2.06-2.20) | 31.15 (28.84-33.45) | 14.59 (13.02-16.17) |
| 8 | 2017-2020 | 6,811 | 4,113 | 6,640,057 | <0.0001 | 1.65 (1.61-1.69) | 25.64 (24.43-26.86) | 15.49 (14.54-16.43) |
| 9 | 2016-2020 | 10,834 | 7,585 | 10,406,868 | <0.0001 | 1.42 (1.40-1.45) | 20.82 (19.94-21.70) | 14.58 (13.84-15.31) |
| 10 | 2014-2020 | 3,699 | 1,965 | 1,874,708 | <0.0001 | 1.88 (1.82-1.94) | 28.19 (25.78-30.59) | 14.97 (13.22-16.73) |
| 11 | 2016-2020 | 1,854 | 1,028 | 1,409,092 | <0.0001 | 1.80 (1.72-1.88) | 26.31 (23.64-28.99) | 14.59 (12.60-16.59) |
| 12 | 2014-2020 | 1,138 | 540 | 536,976 | <0.0001 | 2.10 (1.99-2.23) | 30.28 (25.62-34.93) | 14.37 (11.16-17.57) |
| 13 | 2009-2016 | 1,556 | 880 | 753,072 | <0.0001 | 1.76 (1.67-1.86) | 25.83 (22.20-29.46) | 14.61 (11.88-17.34) |
| 14 | 2013-2020 | 2,864 | 1,968 | 1,633,243 | <0.0001 | 1.45 (1.40-1.50) | 21.92 (19.65-24.19) | 15.06 (13.18-16.94) |
| 15 | 2009-2016 | 3,333 | 2,401 | 2,044,962 | <0.0001 | 1.38 (1.34-1.43) | 20.37 (18.42-22.33) | 14.68 (13.02-16.34) |
| 16 | 2018-2020 | 723 | 404 | 873,527 | <0.0001 | 1.79 (1.66-1.91) | 27.59 (24.11-31.07) | 15.42 (12.81-18.02) |
| 17 | 2018-2020 | 3,052 | 2,366 | 5,130,888 | <0.0001 | 1.29 (1.24-1.33) | 19.83 (18.61-21.05) | 15.37 (14.30-16.44) |
| 18 | 2017-2020 | 3,551 | 2,859 | 4,928,714 | <0.0001 | 1.24 (1.20-1.28) | 18.01 (16.83-19.20) | 14.50 (13.44-15.56) |
| 19 | 2013-2020 | 708 | 476 | 393,266 | <0.0001 | 1.48 (1.36-1.59) | 22.50 (17.82-27.19) | 15.13 (11.29-18.97) |
| 20 | 2014-2020 | 1,749 | 1,376 | 1,279,907 | <0.0001 | 1.27 (1.20-1.32) | 19.52 (17.10-21.94) | 15.36 (13.21-17.51) |
| 21 | 2014-2020 | 553 | 370 | 356,053 | <0.0001 | 1.49 (1.37-1.62) | 22.19 (17.29-27.08) | 14.85 (10.84-18.85) |

| Cluster | Period | Observed | Expected | Population | P Value | RR (95% CI) | Inside Rate (95% CI) | Outside Rate (95% CI) |
|---|---|---|---|---|---|---|---|---|
| 22 | 2017-2020 | 509 | 338 | 576,312 | <0.0001 | 1.50 (1.38-1.64) | 22.08 (18.24-25.92) | 14.66 (11.54-17.79) |
| 23 | 2011-2018 | 85 | 38 | 32,461 | 0.0001 | 2.24 (1.79-2.74) | 32.73 (13.05-52.41) | 14.63 (1.47-27.79) |
| 24 | 2013-2020 | 202 | 124 | 107,197 | 0.0003 | 1.627 (1.41-1.86) | 23.55 (14.37-32.74) | 14.46 (7.26-21.66) |
| 25 | 2005-2008 | 382 | 274 | 491,559 | 0.001 | 1.39 (1.24-1.53) | 19.43 (15.53-23.32) | 13.94 (10.64-17.24) |
| 26 | 2017-2020 | 165 | 105 | 173,279 | 0.048 | 1.57 (1.33-1.81) | 23.81 (16.54-31.07) | 15.15 (9.35-20.94) |

***Supplementary Table 4***. SaTScan results for the Black population, including the time period of the cluster, the number of SUD-related deaths observed, the number of SUD-related deaths expected, the total population within the cluster, the estimated relative risk (RR) with their corresponding 95% confidence interval (CI), the mortality rate inside and outside the identified cluster with their corresponding 95% CI.

| Cluster | Period | Observed | Expected | Population | P Value | RR (95% CI) | Inside Rate (95% CI) | Outside Rate (95% CI) |
|---|---|---|---|---|---|---|---|---|
| 1 | 2016-2020 | 9,959 | 3,263 | 5,134,050 | <0.0001 | 3.050 (2.980-3.110) | 38.80 (37.09-40.50) | 12.71 (11.74-13.69) |
| 2 | 2016-2020 | 9,143 | 3,294 | 5,025,972 | <0.0001 | 2.770 (2.720-2.830) | 36.38 (34.72-38.05) | 13.11 (12.11-14.11) |
| 3 | 2016-2020 | 1,387 | 260 | 411,382 | <0.0001 | 5.370 (5.070-5.620) | 67.43 (59.50-75.37) | 12.64 (9.20-16.08) |
| 4 | 2016-2020 | 1,247 | 496 | 732,201 | <0.0001 | 2.510 (2.370-2.640) | 34.06 (29.83-38.29) | 13.55 (10.88-16.21) |
| 5 | 2017-2020 | 1,519 | 676 | 1,216,636 | <0.0001 | 2.240 (2.130-2.360) | 31.21 (28.07-34.35) | 13.89 (11.80-15.99) |
| 6 | 2016-2020 | 881 | 322 | 484,106 | <0.0001 | 2.730 (2.570-2.920) | 36.40 (31.02-41.77) | 13.30 (10.05-16.55) |
| 7 | 2016-2020 | 868 | 323 | 438,123 | <0.0001 | 2.690 (2.520-2.870) | 39.62 (33.73-45.52) | 14.74 (11.15-18.34) |
| 8 | 2013-2020 | 1,678 | 865 | 842,290 | <0.0001 | 1.930 (1.850-2.030) | 28.46 (24.86-32.06) | 14.67 (12.08-17.26) |
| 9 | 2018-2020 | 1,059 | 463 | 1,068,447 | <0.0001 | 2.280 (2.150-2.420) | 33.04 (29.59-36.49) | 14.44 (12.17-16.72) |
| 10 | 2018-2020 | 2,232 | 1,411 | 3,526,988 | <0.0001 | 1.580 (1.510-1.640) | 21.09 (19.58-22.61) | 13.34 (12.13-14.54) |
| 11 | 2015-2020 | 565 | 255 | 299,066 | <0.0001 | 2.210 (2.030-2.400) | 31.49 (25.13-37.85) | 14.21 (9.94-18.48) |
| 12 | 2018-2020 | 280 | 107 | 249,025 | <0.0001 | 2.620 (2.320-2.920) | 37.48 (29.88-45.08) | 14.32 (9.62-19.02) |
| 13 | 2018-2020 | 1,011 | 672 | 1,663,632 | <0.0001 | 1.500 (1.410-1.600) | 20.26 (18.09-22.42) | 13.46 (11.70-15.23) |
| 14 | 2018-2020 | 362 | 206 | 516,131 | <0.0001 | 1.750 (1.560-1.940) | 23.38 (19.21-27.55) | 13.30 (10.16-16.45) |
| 15 | 2017-2020 | 269 | 148 | 288,017 | <0.0001 | 1.810 (1.610-2.040) | 23.35 (17.77-28.93) | 12.85 (8.71-16.99) |
| 16 | 2018-2020 | 962 | 716 | 1,647,984 | <0.0001 | 1.340 (1.250-1.430) | 19.46 (17.33-21.59) | 14.48 (12.64-16.32) |
| 17 | 2018-2020 | 42 | 10 | 23,633 | <0.0001 | 4.170 (2.880-5.460) | 59.24 (28.21-90.27) | 14.10 (-1.04-29.25) |

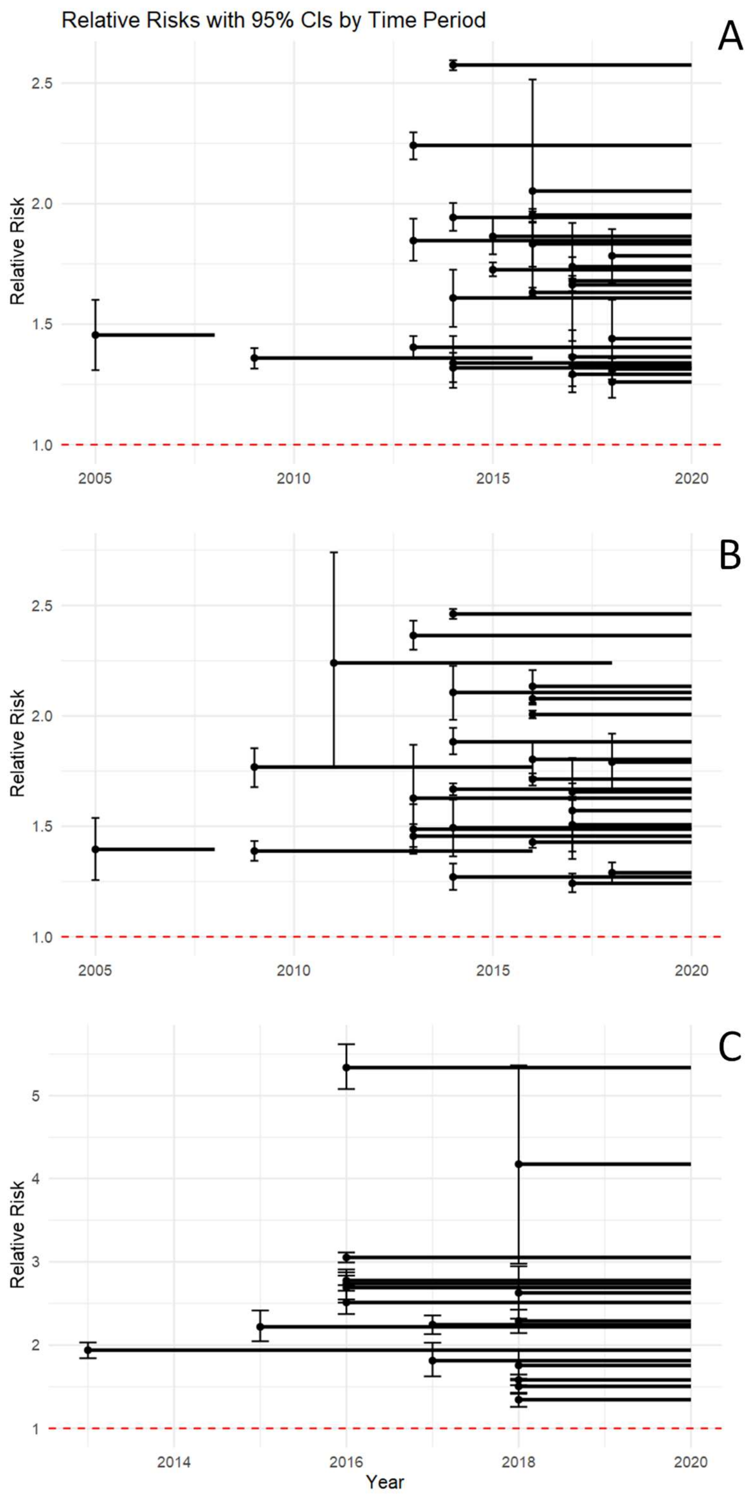


***Supplementary Figure 3***. Relative risk (RR) and length duration of each of the clusters identified for A) total population, B) White population, and C) Black population.

### *2.3. Sensitivity Analysis and Misclassification Rates*

The results from our sensitivity analyses demonstrate high stability and consistency in cluster detection across different simulation settings, reinforcing the robustness of our findings. First, we evaluated the stability of clusters across three levels of Monte Carlo replications: Standard (999), Intermediate (4,999), and High (9,999). For the general population (Supplementary Figure 4) and the Black population (Supplementary Figure 6), the number and location of clusters remained stable across all replication levels. The White population showed minor variation, with a cluster in southeastern New Mexico detected only with the Standard replication setting (Supplementary Figure 5). Contingency tables (Supplementary Tables 4 to 6) show zero misclassification for the general population and Black population analyses across all replication levels, while the White population exhibited a slight increase in misclassification with higher replications, confirming the robustness of cluster detection for the general population and most subpopulations.

Our second sensitivity analysis excluded counties with exceptionally high SUD-related death counts, those above the 98th percentile, to assess the effect of high-count outliers on cluster detection. Figures S7 to S9 illustrate that clusters identified with and without these outliers remained largely consistent, though minor displacements occurred. The exclusion of outliers tested the resilience of clusters by ensuring that RR estimates were not artificially inflated by a few areas with extremely high mortality rates. Misclassification rates in the outlier-excluded dataset (Supplementary Table 8) were low for the general population (15%) and somewhat higher for the White (28%) and Black (36%) populations, consistent with expectations given the smaller sizes and increased variability within these subgroups.

The misclassification rates provide additional context for interpreting the model's performance and potential overdispersion effects. Lower rates for the general population analysis suggest that cluster detection accuracy is high, reflecting stable patterns across a large dataset with broad geographic coverage. In contrast, the relatively higher misclassification rates for White and Black populations indicate greater sensitivity in subgroup analyses due to smaller sample sizes and geographic heterogeneity. These rates highlight the effect of extra-Poisson variability on cluster boundary precision within subpopulations, a common occurrence in epidemiological analyses of diverse demographic groups.

In summary, the stability of clusters across various replication levels and consistency in outlier-excluded datasets support the reliability of our primary findings. By confirming that cluster detection is unaffected by extreme values and is stable across simulation parameters, these sensitivity analyses underscore the robustness of our spatial scan approach for analyzing SUD mortality and help minimize potential biases associated with scan statistics in epidemiological studies.

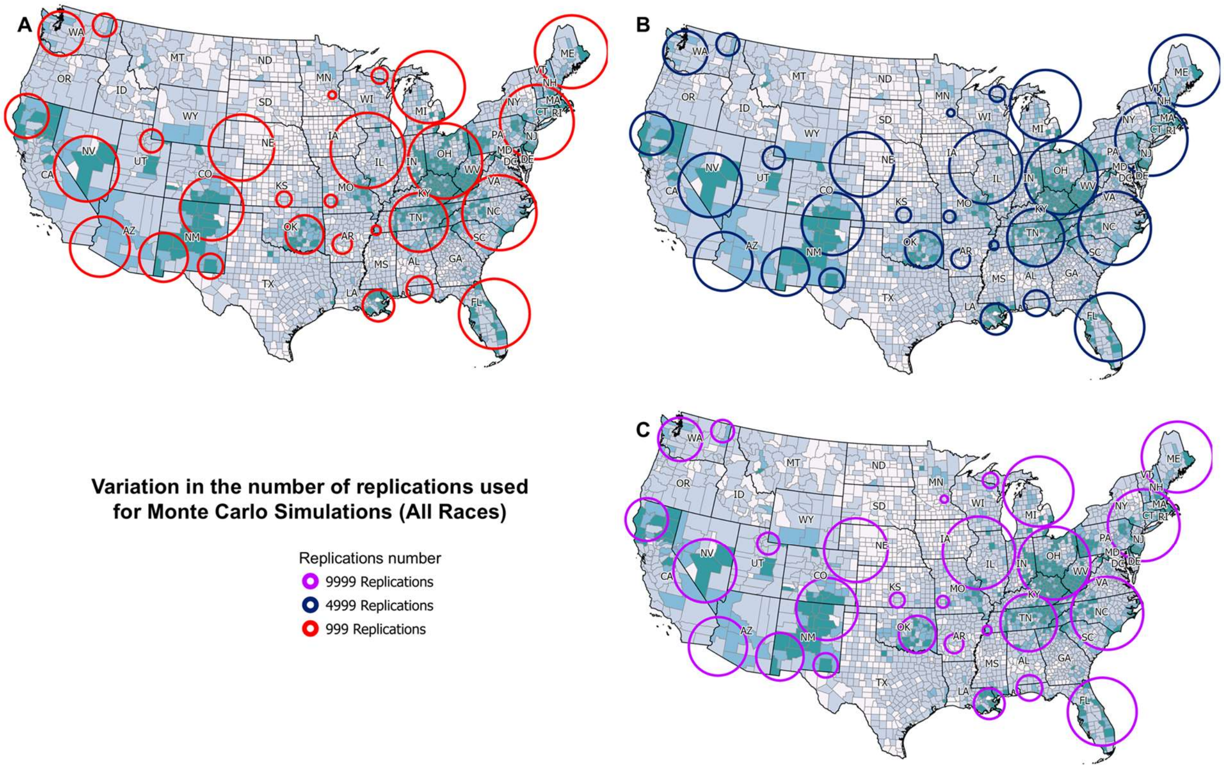


***Supplementary Figure 4***. Variation of the Monte Carlo simulations for the total population. In A) default 999 simulations, B) 4999 simulations, and C) 9999 simulations

***Supplementary Table 4***. Monte Carlo Sensitivity Results. Contingency tables comparing the results for the different number of simulations in the total population: the number of counties classified as cluster, classified as outside of clusters, and classified as cluster both in the analysis performed with the Standard number of replications and with the corresponding variation in the number of replications.

| **Monte Carlo Variations Total population** | | | |
|---|---|---|---|
| | Standard | **Intermediate** | **High** |
| ***Cluster*** | 1301 | 1301 | 1301 |
| ***No Cluster*** | 1807 | 1807 | 1807 |
| ***Also cluster in Standard*** | 1301 | 1301 | 1301 |
| ***Misclassification Rate*** | 0 | 0 | 0 |

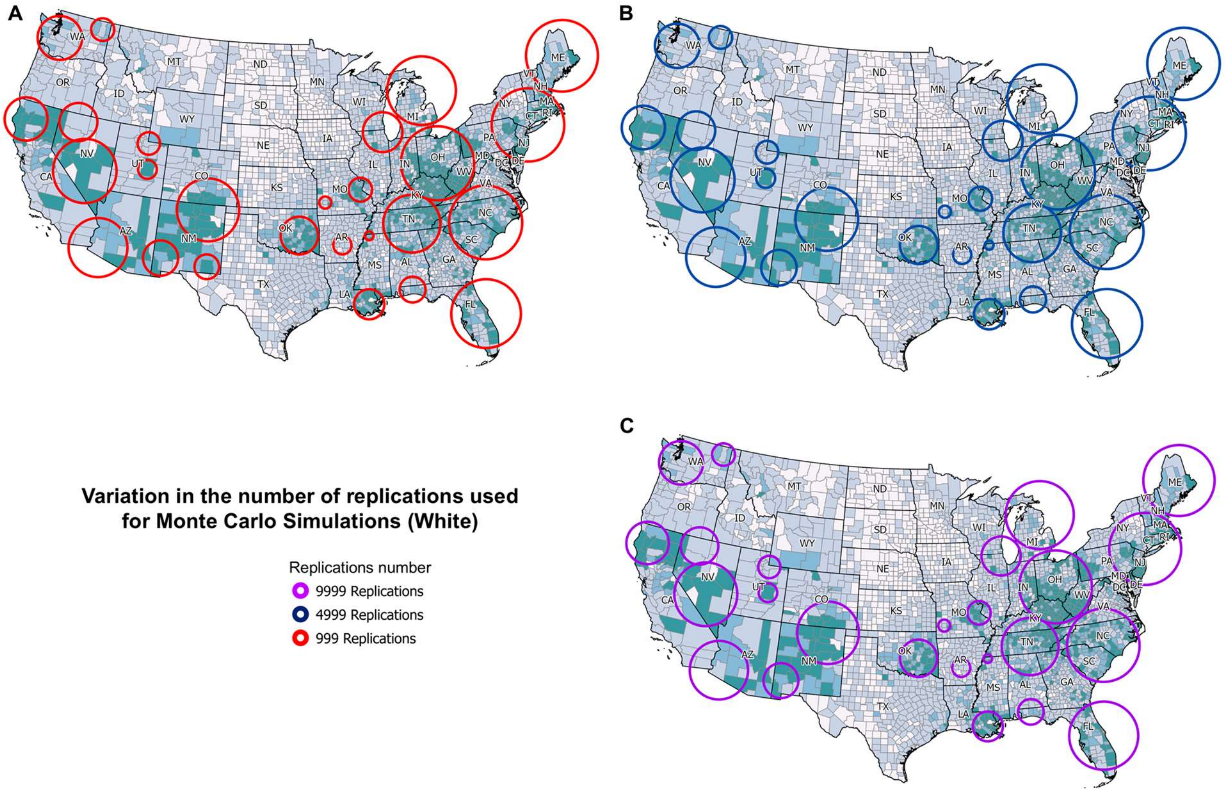


***Supplementary Figure 5***. Variation of the Monte Carlo simulations for the White population. In A) default 999 simulations, B) 4999 simulations, and C) 9999 simulations

***Supplementary Table 5***. Monte Carlo Sensitivity Results. Contingency tables comparing the results for the different number of simulations in the total White population: the number of counties classified as cluster, classified as outside of clusters, and classified as cluster both in the analysis performed with the Standard number of replications and with the corresponding variation in the number of replications.

| **Monte Carlo Variations White Population** | | | |
|---|---|---|---|
| | **Standard** | **Intermediate** | **High** |
| ***Cluster*** | 1076 | 1073 | 1073 |
| ***No Cluster*** | 2032 | 2035 | 2035 |
| ***Also cluster in Standard*** | 1076 | 3 | 3 |
| ***Misclassification Rate*** | 0 | 0.0028 | 0.0028 |

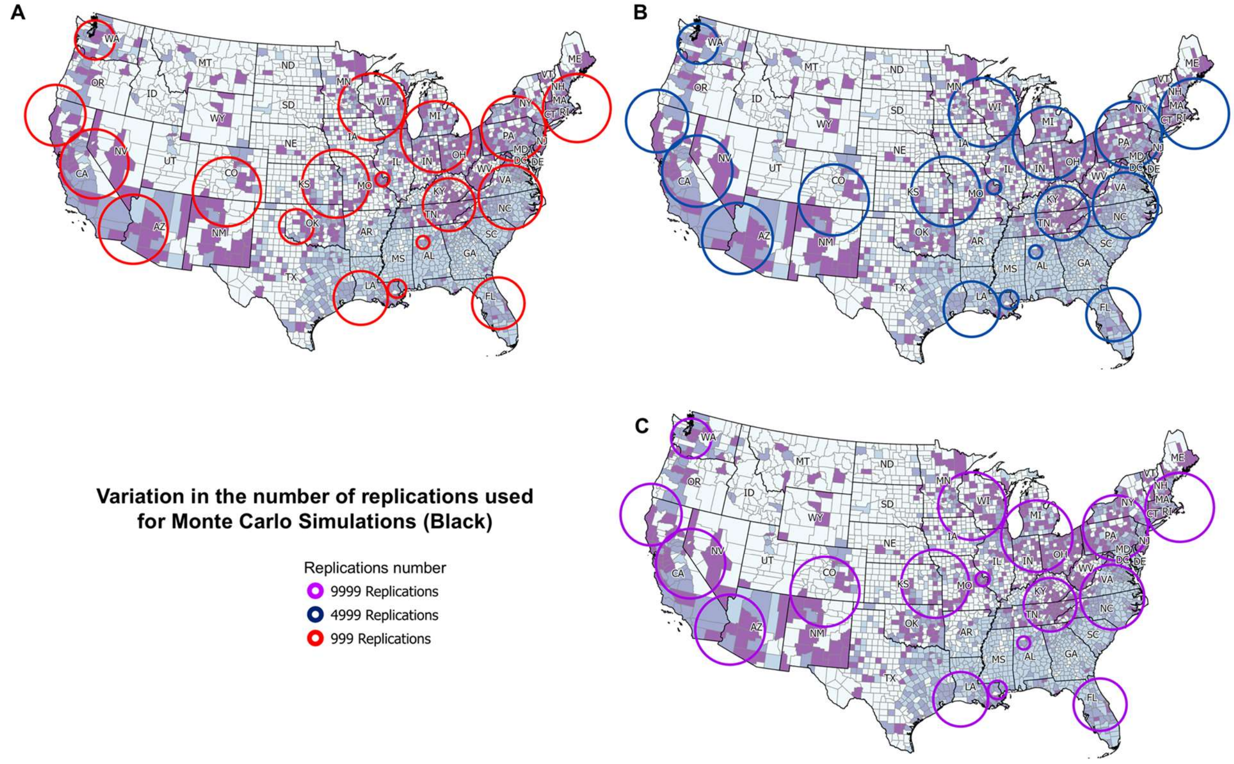


***Supplementary Figure 6***. Variation of the Monte Carlo simulations for the Black population. In A) default 999 simulations, B) 4999 simulations, and C) 9999 simulations

***Supplementary Table 6***. Monte Carlo Sensitivity Results. Contingency tables comparing the results for the different number of simulations in the Black population: the number of counties classified as cluster, classified as outside of clusters, and classified as cluster both in the analysis performed with the Standard number of replications and with the corresponding variation in the number of replications.

| **Monte Carlo Variations Black Population** | | | |
|---|---|---|---|
| | **Standard** | **Intermediate** | **High** |
| ***Cluster*** | 1263 | 1263 | 1263 |
| ***No Cluster*** | 1845 | 1845 | 1845 |
| ***Also cluster in Standard*** | 1263 | 1263 | 1263 |
| ***Misclassification Rate*** | 0 | 0 | 0 |

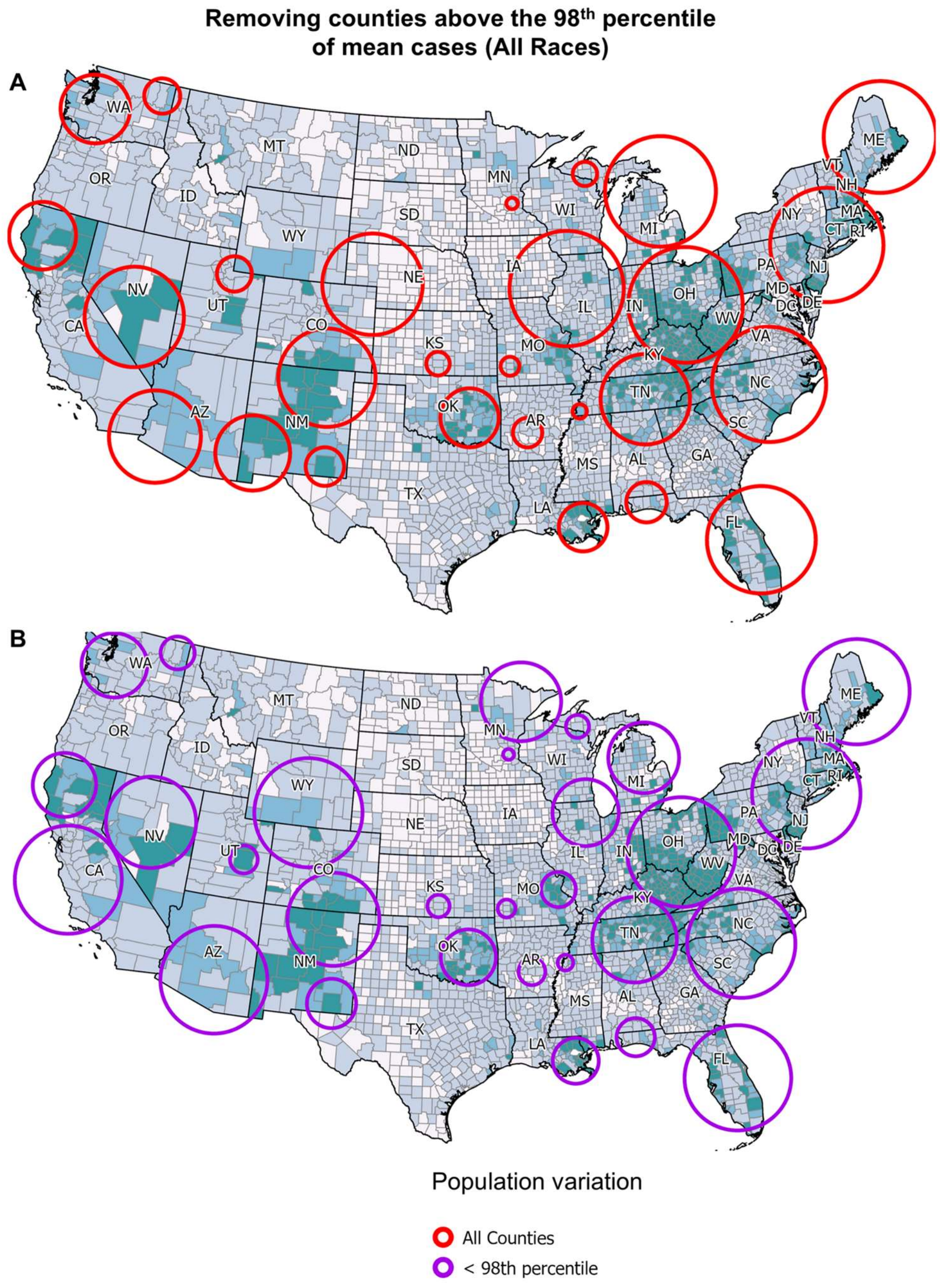


***Supplementary Figure 7***. Removing the counties considered mortality outliners, whose mean SUD-related death counts over the whole study period greater than the 98th percentile for the total population. In A) the original result without the removal process. In B) the results after the removal process.

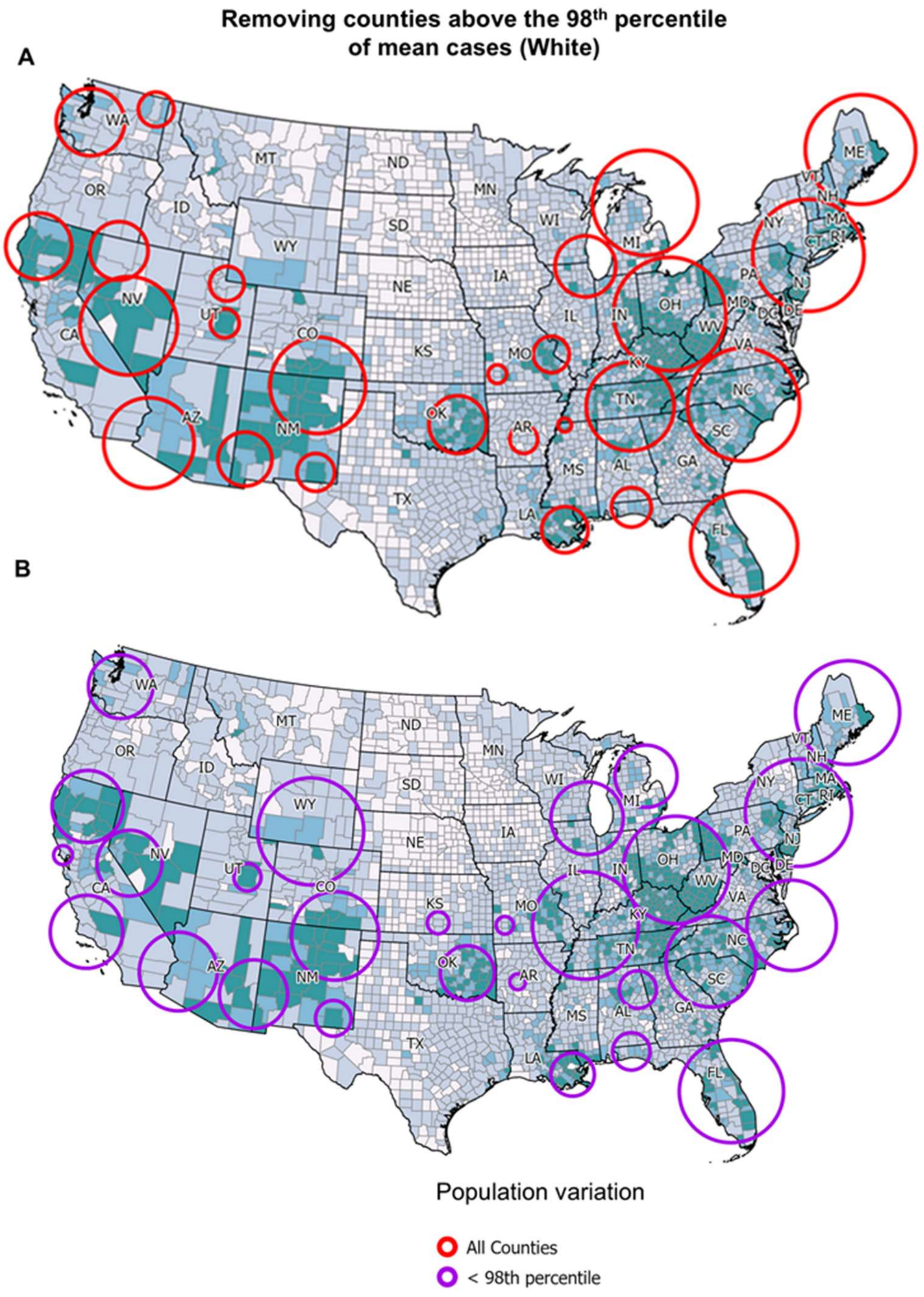


***Supplementary Figure 8***. Outlier Exclusion Results. Outlier Exclusion Results. Removing the counties considered mortality outliners, whose mean SUD-related death counts over the whole study period greater than the 98th percentile for the White population. In A) the original result without the removal process. In B) the results after the removal process.

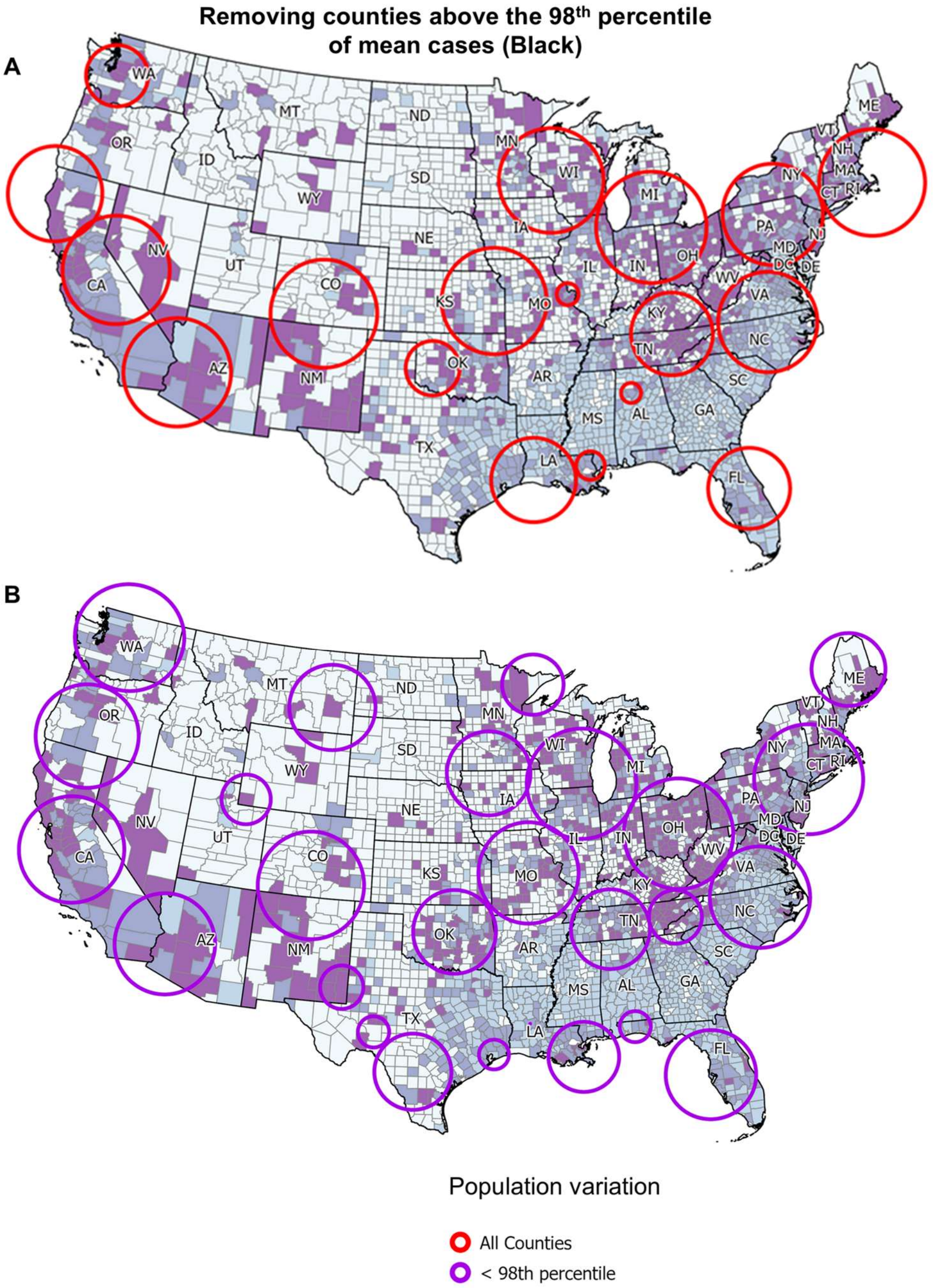


***Supplementary Figure 9***. Outlier Exclusion Results. Removing the counties considered mortality outliners, whose mean SUD-related death counts over the whole study period greater than the 98th percentile for the Black population. In A) the original result without the removal process. In B) the results after the removal process.

***Supplementary Table** 7*. Outlier Exclusion Results. Contingency tables comparing the results for the different number of simulations in all populations: the number of counties classified as cluster, classified as outside of clusters, and classified as cluster both in the analysis performed with the original dataset and after removing counties in the >98th.

| **Control for outliners (counties >98th percentile)** | | | | | | |
|---|---|---|---|---|---|---|
| | **All Races** | | **White** | | **Black** | |
| | *Original* | *>98th percentile* | *Original* | *>98th percentile* | *Original* | *>98th percentile* |
| ***Cluster*** | 1301 | 1177 | 1076 | 1258 | 1263 | 1486 |
| ***No Cluster*** | 1807 | 1931 | 2032 | 1850 | 1845 | 1622 |
| ***Also cluster in original*** | 1301 | 1004 | 1076 | 906 | 1263 | 949 |
| ***Misclassification Rate*** | 0 | 0.15 | 0 | 0.28 | 0 | 0.36 |

## 3. SUPPLEMETARY LIMITATIONS OF THE STUDY

This study has two primary limitations. First, relying on mortality data classified under specific ICD-10 codes for unintentional drug poisoning as a proxy for estimating SUD mortality introduces potential inaccuracies. Cases where the substance implicated in death is not accurately identified by the medical examiner may be missed in our dataset. This underscores the need for improved quality in characterizing SUD-related deaths and standardizing substance classification across jurisdictions, including explicit coding for emerging substances like fentanyl.

Second, the ecological and longitudinal nature of our analysis means that while our findings indicate broader trends, they may not uniformly apply across all scales due to the multifaceted factors influencing the SUD epidemic. Our exploratory approach, using spatial scan statistics to elucidate the spatiotemporal dynamics of SUD mortality, highlights general patterns but also necessitates further investigation into substance-specific clusters. A more detailed analysis, segmented by substance and focused on specific geographical and temporal segments, is crucial for understanding how specific substances contribute to the emergence and distribution of SUD mortality clusters. This comprehensive perspective is essential for tailoring interventions and policy responses to the complex landscape of the SUD epidemic.

Third, the spatiotemporal clustering analysis employed in this study was specifically designed to identify clusters that emerge, persist, or disappear over time, reflecting the dynamic nature of the SUD epidemic. By using Kulldorff's spatial scan statistic within the SaTScan software, we applied a space-time permutation model that is sensitive to temporal and spatial variations in mortality rates. This method allows us to capture shifting clusters, which may be influenced by fluctuations in drug availability, shifts in trafficking routes, policy changes, and healthcare interventions over time. The absence of consistently persistent clusters across the study period suggests that these external factors might be driving changes in the epidemic's geographic epicenter. Therefore, we acknowledge that variability in data reporting practices and regional differences in healthcare access and socioeconomic conditions may also contribute to the observed patterns. These factors were not included in this study and warrant further investigation.

Likewise, while our analysis identified distinct clustering patterns by racial groups, a detailed examination of the confounding effects between race and urban-rural status was beyond the scope of this study. Instead, we focused on conducting individual racial analyses to highlight disparities in cluster formation across subpopulations. Our findings showed that rural clusters were more prevalent among the White population, particularly in the West, while the Black population was more frequently associated with urban clusters in the East. Future studies could explore these interactions further to understand the complex dynamics of race and geographic status in the SUD epidemic, incorporating socioeconomic and healthcare access factors for a more comprehensive analysis.

Fourth, one key consideration relates to potential bias in RR estimates due to cluster selection based on statistical significance, as highlighted by Prates et al. (2014). While our study benefits from a large sample size, extended temporal coverage, and significant statistical power, factors which mitigate RR bias, there remains the possibility of upward bias in smaller clusters with fewer cases. Sensitivity analyses involving varying simulation parameters and removing outliers

supported the robustness of our findings, and demonstrated robustness across various sensitivity analyses, reinforcing the reliability of identified high-risk clusters in SUD-related mortality. Cluster stability remained consistent for the general and Black populations under varying Monte Carlo replications (999, 4,999, and 9,999), with no variation in misclassification rates. Outlier exclusion, removing counties above the 98th percentile in SUD deaths, further supported this stability, with minimal spatial shifts in clusters across all populations. This consistency suggests that our clusters reflect genuine SUD mortality patterns, unaffected by extreme values or simulation settings.

However, despite these robust findings, our results should be interpreted with caution due to potential limitations inherent in the use of scan statistics, particularly in subgroup analyses. Overdispersion, extra-Poisson variability resulting from unmodeled heterogeneity, presents an important consideration in this context. The higher misclassification rates observed in White and Black subpopulations compared to the general population suggest some degree of overdispersion, which could impact the precision of RR estimates for these groups. This overdispersion likely arises from geographic, socioeconomic, and demographic diversity within subpopulations that the Poisson model in SaTScan may not fully capture. Therefore, we advise caution in interpreting our results and recognize that alternative models may be explored in future studies to address this limitation further.

## 4. REFERENCES


1. M. K. A spatial scan statistic. *Communications in Statistics: Theory and Methods* 1997; **26**: 1481-96.
2. Wand H, Ramjee G. Targeting the hotspots: investigating spatial and demographic variations in HIV infection in small communities in South Africa. *Journal of the International AIDS Society* 2010; **13**(1): 41.
3. Ryan J, Mbui J, Rashid J, et al. Spatial clustering and epidemiological aspects of visceral Leishmaniasis in two endemic villages, Baringo District, Kenya. *American Journal of Tropical Medicine and Hygiene* 2006; **74**(2): 308 - 17.
4. Kulldorf M, Song C, Gregoria D, Samociuk H, DeChello L. Cancer map patterns: are they random or not? *Am J Prev Med* 2006; **30**: s37 - s49.
5. Cuadros D, Awad S, Abu-Raddad L. Mapping HIV clustering: a strategy for identifying populations at high risk of HIV infection in sub-Saharan Africa. *International Journal of Health Geographics* 2013; **12**(1): 28.
6. Jones P, Gunnell D, Platt S, et al. Identifying probable suicide clusters in Wales using national mortality data. *PLoS One* 2013; **8**(8): e71713.
7. Malleson N, Andresen MA. Spatio-temporal crime hotspots and the ambient population. *Crime science* 2015; **4**(1): 1-8.
8. Ruiz-Grosso P, Miranda JJ, Gilman RH, et al. Spatial distribution of individuals with symptoms of depression in a periurban area in Lima: an example from Peru. *Annals of epidemiology* 2016; **26**(2): 93-9. e2.
9. Cordes J. Spatial Trends in Opiate Overdose Death in North Carolina: 1999-2015. 2017.
10. Brownstein JS, Green TC, Cassidy TA, Butler SF. Geographic information systems and pharmacoepidemiology: using spatial cluster detection to monitor local patterns of prescription opioid abuse. *Pharmacoepidemiology and drug safety* 2010; **19**(6): 627-37.
11. Hester L, Shi X, Morden N. Characterizing the geographic variation and risk factors of fatal prescription opioid poisoning in New Hampshire, 2003–2007. *Annals of GIS* 2012; **18**(2): 99-108.
12. Hernandez A BA, Li J, MacKinnon NJ, Hincapie AL, Cuadros DF. Epidemiological and geospatial

profile of the prescription opioid crisis in Ohio, United States. *Scientific Reports* 2020; **10**(1): 4341.
13. Hernández A LM, MacKinnon NJ, Branscum AJ, Cuadros DF. "Know your epidemic, know your response": Epidemiological assessment of the substance use disorder crisis in the United States. *PLoS One* 2021; **May 26;16(5):e0251502.**
14. Amin RW, Fritsch BA, Retzloff JE. Spatial clusters of breast cancer mortality and incidence in the contiguous USA: 2000–2014. *Journal of General Internal Medicine* 2019; **34**: 412-9.
15. Amin RW, Stafford B, Guttmann RP. A spatial study of bladder cancer mortality and incidence in the contiguous US: 2000–2014. *Science of the total environment* 2019; **670**: 806-13.
16. Chen H, Zeng D, Yan P, Chen H, Zeng D, Yan P. Data analysis and outbreak detection. *Infectious Disease Informatics: Syndromic Surveillance for Public Health and BioDefense* 2010: 49-72.

17. Ulrich SE, Sugg MM, Desjardins MR, Runkle JD. Disparities in spatiotemporal clustering of maternal mental health conditions before and during the COVID-19 pandemic. *Health & Place* 2024; **89**: 103307.
18. Ulrich SE, Sugg MM, Ryan SC, Runkle JD. Mapping high-risk clusters and identifying place-based risk factors of mental health burden in pregnancy. *SSM-Mental Health* 2023; **4**: 100270.
19. Amin R, Guttmann RP, Rivera-Muñiz B, Holley M, Uher M. Spatial and space-time clusters of suicides in the contiguous USA (2000–2019). *Annals of epidemiology* 2022; **76**: 150-7.
